# Supplementary figures and images for: Effects of an mHealth intervention for community health workers on maternal and child nutrition and health service delivery in India: protocol for a quasi-experimental mixed-methods evaluation
Source: BMJ Open. 2019 Mar 27;9(3):e025774. doi: 10.1136/bmjopen-2018-025774 (PMC6475202; doi:10.1136/bmjopen-2018-025774)

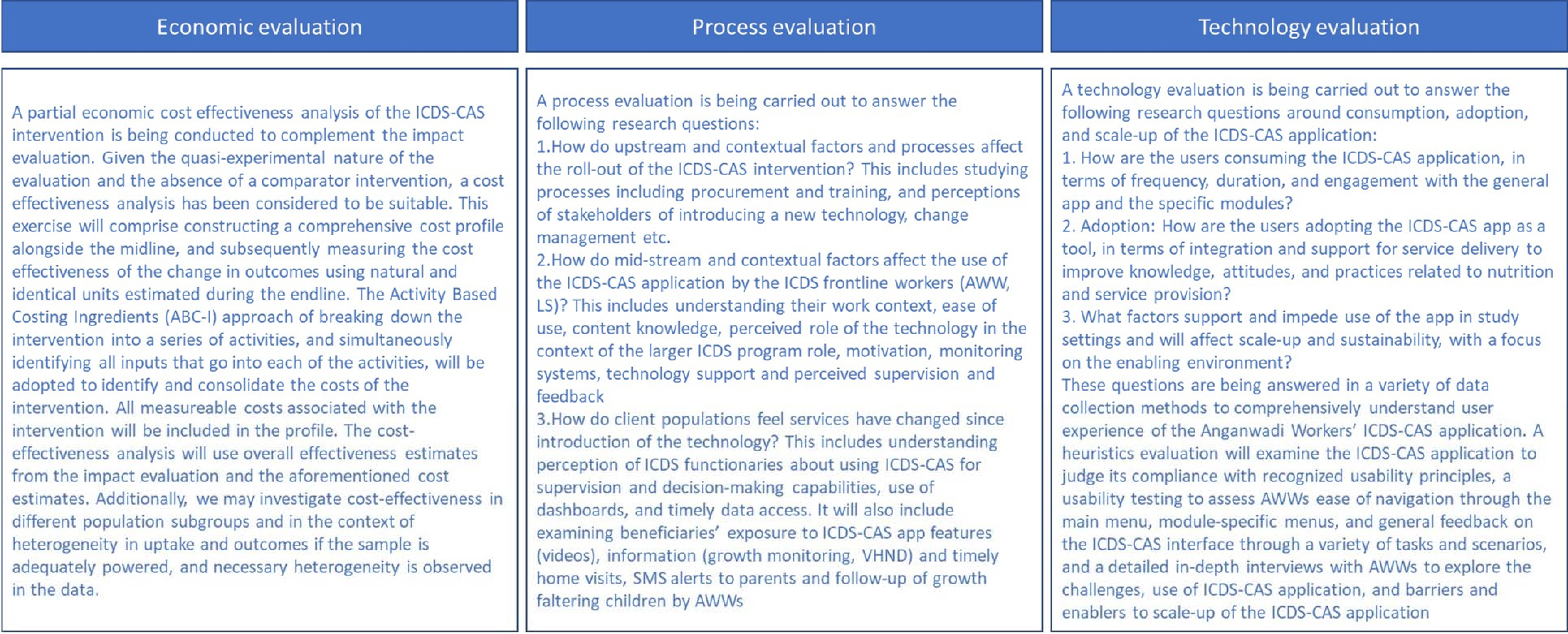

Supplement: Supplementary data [file bmjopen-2018-025774supp001.jpg]
