# Supplementary material for: Effects of an mHealth intervention for community health workers on maternal and child nutrition and health service delivery in India: protocol for a quasi-experimental mixed-methods evaluation
Source: BMJ Open. 2019 Mar 27;9(3):e025774. doi: 10.1136/bmjopen-2018-025774 (PMC6475202; doi:10.1136/bmjopen-2018-025774)
Supplement: Supplementary data [file bmjopen-2018-025774supp002.pdf]

Population, Health and Nutrition Related Indicators from National Family Health Survey (NFHS): India, Madhya Pradesh and Bihar, 2015-16

NFHS Indicators	India	Madhya Pradesh	Bihar
Sex ratio of the total population (females per 1,000 males)	1009	955	1075
Children under age 5 years whose birth was registered (%)	76.1	78.4	60.3
Households with electricity (%)	83.2	86.4	54.1
Households using clean fuel for cooking (%)	43.8	29.6	17.8
Households with an improved drinking-water source (%)	89.3	79.5	98.2
Households using improved sanitation facility (%)	36.7	19.4	20.7
Population (female) age 6 years and above who ever attended school (%)	68.8	64.0	56.9
Women with 10 or more years of schooling (%)	27.3	14.1	19.5
Women age 15-19 years who were ever pregnant (%)	9.2	8.6	12.8
Under-five mortality rate (U5MR)	56	69	60
Married women use of family planning methods (%)	51.7	51.3	22.6
Health worker ever talked to female non-users about family planning (%)	17.2	19.3	11.6
Mothers who consumed iron folic acid for 100+ days during pregnancy (%)	25.9	20.5	9.4
Mothers who had full antenatal care (%)	16.7	8.3	3.0
Institutional births (%)	75.1	76.4	62.7
Institutional births in public facility (%)	54.4	70.3	48.2
Children age 12-23 months fully immunized (%)	61.3	50.2	61.9
Children under age 6 months exclusively breastfed (%)	56.0	59.6	54.2
Total children age 6-23 months receiving an adequate diet (%)	8.8	6.0	7.4
Children under 5 years who are stunted (height-for-age) (%)	41.2	43.6	49.3
Children under 5 years who are wasted (weight-for-height) (%)	21.5	27.1	20.8
Children under 5 years who are underweight (weight-for-age) (%)	38.3	45.0	44.6
Women whose Body Mass Index (BMI) is below normal (<18.5 kg/m2) (%)	26.7	31.8	31.8
Children age 6-59 months who are anaemic (<11 g/dl) (%)	59.4	69.9	64.0
Pregnant women age 15-49 years who are anaemic (<11 g/dl) (%)	52.1	56.4	58.0

Source: NFHS-4 fact sheets for India, Madhya Pradesh and Bihar (http://rchiips.org/NFHS/factsheet_NFHS-4.shtml)
