# Supplementary material for: Effects of an mHealth intervention for community health workers on maternal and child nutrition and health service delivery in India: protocol for a quasi-experimental mixed-methods evaluation
Source: BMJ Open. 2019 Mar 27;9(3):e025774. doi: 10.1136/bmjopen-2018-025774 (PMC6475202; doi:10.1136/bmjopen-2018-025774)
Supplement: Supplementary data [file bmjopen-2018-025774supp003.pdf]

Balance in Covariates at Anganwadi, Household and Individual Beneficiary levels

Indicators	Bihar					Madhya Pradesh				
	N	Mean (Comp-arison)	Mean (Interv-ention)	Mean Diff. (Std. Err.)		N	Mean (Comp-arison)	Mean (Interv-ention)	Mean Diff. (Std. Err.)	
				District Pairs	Village Pairs				District Pairs	Village Pairs
**Respondent Characteristics**										
Average age in completed years	3143	24.31	24.90	0.587*** (0.18)	0.583*** (0.126)	2636	24.18	24.12	-0.065 (0.151)	-0.074 (0.118)
Average education level in years	3143	3.96	3.30	-0.663*** (0.231)	-0.695*** (0.17)	2636	4.55	4.92	0.353* (0.198)	0.35** (0.153)
% of respondents who work to earn income	3143	0.11	0.12	0.014 (0.015)	0.017 (0.011)	2636	0.45	0.44	-0.017 (0.02)	-0.018 (0.014)
% of respondents who have an Aadhaar card	3143	0.97	0.96	-0.015** (0.006)	-0.016*** (0.005)	2636	0.94	0.94	-0.002 (0.01)	-0.004 (0.008)
% of respondents who own a bank account	3143	0.77	0.80	0.029* (0.017)	0.026** (0.013)	2636	0.78	0.81	0.026 (0.019)	0.024* (0.014)
% of respondents who are currently married	3143	1.00	1.00	-0.001 (0.002)	-0.001 (0.002)	2636	0.99	0.99	0 (0.003)	0.001 (0.002)
% of respondents who were residing in their permanent marital home at the time of survey	3143	0.88	0.89	0.012 (0.013)	0.012 (0.009)	2636	0.92	0.9	-0.019 (0.012)	-0.017* (0.009)
% of respondents who were residing in their permanent maternal home at the time of survey	3143	0.02	0.01	-0.011** (0.005)	-0.011*** (0.004)	2636	0.01	0.01	0.002 (0.004)	0.002 (0.003)
**Household Socio-economics**										
% of households which belong to Scheduled Castes or Scheduled Tribe categories	3143	0.35	0.38	0.03 (0.03)	0.031 (0.023)	2636	0.56	0.5	-0.061** (0.028)	-0.06*** (0.021)

Indicators	Bihar					Madhya Pradesh				
	N	Mean (Comparison)	Mean (Intervention)	Mean Diff. (Std. Err.)		N	Mean (Comparison)	Mean (Intervention)	Mean Diff. (Std. Err.)	
				District Pairs	Village Pairs				District Pairs	Village Pairs
% of households which belong to Below-Poverty-Line category	3143	**0.58**	**0.62**	0.039* (0.022)	0.043** (0.017)	2636	0.63	0.66	0.024 (0.022)	0.023 (0.017)
% of households with pucca construction	3143	0.38	0.37	-0.007 (0.024)	-0.009 (0.017)	2636	0.26	0.3	0.036 (0.024)	0.033* (0.018)
% of households with fewer than two rooms for sleeping	3143	**0.34**	**0.41**	0.068*** (0.022)	0.067*** (0.016)	2636	0.39	0.41	0.017 (0.023)	0.022 (0.017)
% of households that own agricultural land	3143	0.47	0.42	-0.051** (0.026)	-0.055*** (0.019)	2636	0.76	0.74	-0.021 (0.022)	-0.019 (0.017)
% of households that mainly use improved fuels for cooking	3143	0.17	0.15	-0.024 (0.017)	-0.024* (0.013)	2636	0.1	0.14	0.033** (0.016)	0.032*** (0.011)
% of households with improved drinking water source on premises	3143	**0.61**	**0.55**	-0.053** (0.025)	-0.055*** (0.02)	2636	0.14	0.14	0.001 (0.018)	0.004 (0.013)
% of households with a functional toilet	3143	**0.24**	**0.39**	0.156*** (0.025)	0.152*** (0.019)	2636	**0.52**	**0.46**	-0.061** (0.029)	-0.063*** (0.022)
% of households with any member practicing open defecation	3143	**0.81**	**0.70**	-0.107*** (0.023)	-0.105*** (0.018)	2636	**0.62**	**0.71**	0.086*** (0.027)	0.086*** (0.02)
% of households with piped water	3143	0.01	0.01	0.004 (0.005)	0.004 (0.004)	2636	0.08	0.07	-0.006 (0.017)	-0.006 (0.013)
% of households with a handwashing facility	3143	0.69	0.66	-0.027 (0.029)	-0.032 (0.021)	2636	0.62	0.65	0.025 (0.03)	0.025 (0.022)
**Anganwadi Center (AWC) Coverage**										
Average population in catchment area	419	1111.70	1116.33	4.633 (28.769)	-2.274 (28.894)	406	730.32	857.24	124.258*** (29.916)	123.823*** (30.838)
Average number of families in catchment area	415	172.45	185.66	13.555** (5.458)	12.706** (5.66)	406	126	153.69	26.827*** (5.223)	26.54*** (5.39)

Indicators	Bihar					Madhya Pradesh				
	N	Mean (Comp-arison)	Mean (Interv-ention)	Mean Diff. (Std. Err.)		N	Mean (Comp-arison)	Mean (Interv-ention)	Mean Diff. (Std. Err.)	
				District Pairs	Village Pairs				District Pairs	Village Pairs
Average number of children aged 0-5 months in catchment area	403	10.56	11.10	0.551 (1.12)	0.606 (1.194)	404	5.64	9.32	3.697*** (1.232)	3.655*** (1.269)
Average number of children aged 6-12 months in catchment area	393	17.64	17.26	-0.307 (1.559)	-0.283 (1.757)	400	7.62	9.58	1.973*** (0.698)	1.984*** (0.75)
Average number of children aged 12-24 months in catchment area	387	27.78	27.80	0.208 (2.256)	0.017 (2.446)	398	15	19.98	5.011*** (1.085)	5.34*** (1.164)
Average number of pregnant women in catchment area	417	12.30	12.36	0.053 (0.458)	0.068 (0.464)	406	7.39	7.71	0.306 (0.576)	0.237 (0.604)
Average number of lactating women in catchment area	417	12.05	11.10	-0.948* (0.513)	-0.976* (0.508)	406	5.86	7.36	1.503*** (0.405)	1.444*** (0.43)
**Anganwadi Center (AWC) Characteristics**										
% AWCs with pucca construction	420	0.55	0.52	-0.033 (0.047)	-0.033 (0.047)	412	0.8	0.85	0.04 (0.037)	0.039 (0.039)
% AWCs with an Anganwadi Helper	420	0.91	0.91	-0.004 (0.028)	-0.005 (0.028)	412	0.96	0.97	0.005 (0.019)	0.005 (0.019)
% AWCs with drinking water on the premises	420	0.43	0.46	0.029 (0.049)	0.029 (0.049)	412	0.68	0.58	-0.098** (0.046)	-0.108** (0.048)
% AWCs with a functional toilet on the premises	420	0.11	0.18	0.07** (0.035)	0.062* (0.034)	412	0.39	0.29	-0.1** (0.045)	-0.103** (0.045)
% AWCs with electricity	420	0.16	0.17	0.003 (0.036)	0.005 (0.037)	412	0.21	0.15	-0.055 (0.038)	-0.054 (0.038)
% AWCs with Salter scales to weigh children	420	0.59	0.74	0.151*** (0.044)	0.148*** (0.044)	412	0.96	0.91	-0.048** (0.024)	-0.049* (0.025)
% AWCs with the new WHO Growth Monitoring charts	420	0.28	0.30	0.026 (0.044)	0.024 (0.048)	412	0.69	0.58	-0.108** (0.046)	-0.113** (0.048)
**Anganwadi Worker (AWW) Characteristics**										

Indicators	Bihar					Madhya Pradesh				
	N	Mean (Comp-arison)	Mean (Interv-ention)	Mean Diff. (Std. Err.)		N	Mean (Comp-arison)	Mean (Interv-ention)	Mean Diff. (Std. Err.)	
				District Pairs	Village Pairs				District Pairs	Village Pairs
Average age in completed years	420	39.11	37.54	-1.565** (0.744)	-1.555** (0.756)	412	39.45	40.57	1.124 (0.868)	1.069 (0.888)
Average education level in years	420	11.53	11.81	0.283 (0.201)	0.278 (0.203)	412	10.12	10.86	0.739** (0.334)	0.75** (0.336)
% AWWs who belong to Scheduled Castes or Scheduled Tribe categories	420	0.17	0.21	0.041 (0.038)	0.043 (0.039)	412	0.53	0.43	-0.102*** (0.039)	-0.108** (0.042)
% AWWs who own a bank account	420	0.99	1.00	0.01 (0.009)	0.01 (0.01)	412	1	1	0.005 (0.005)	0.005 (0.005)
% AWWs who are currently married	420	0.93	0.96	0.024 (0.022)	0.024 (0.023)	412	0.82	0.87	0.05 (0.036)	0.049 (0.038)
% AWWs who are presently engaged in any other work to earn additional income	420	0.01	0.06	0.042** (0.018)	0.043** (0.017)	412	0.03	0.12	0.087*** (0.026)	0.088*** (0.026)
% AWWs who received job training on joining	420	0.99	0.97	-0.014 (0.014)	-0.014 (0.014)	382	0.95	0.93	-0.015 (0.025)	-0.017 (0.028)
% AWWs who report ownership of a smart phone	420	0.03	0.93	0.895*** (0.021)	0.895*** (0.021)	412	0.23	0.94	0.713*** (0.034)	0.711*** (0.036)
**% AWWs who report providing the following services in the last calendar month**										
Take Home Ration to pregnant women, lactating women, and children < 3 years	420	0.70	0.19	-0.509*** (0.041)	-0.507*** (0.041)	412	0.98	0.96	-0.014 (0.017)	-0.015 (0.018)
Home Visits	420	0.86	0.89	0.034 (0.031)	0.033 (0.031)	412	0.98	0.98	0 (0.014)	0 (0.014)
Iron supplements to pregnant women	420	0.13	0.30	0.174*** (0.039)	0.172*** (0.04)	412	0.87	0.87	0.006 (0.033)	0 (0.033)
Iron supplements to children aged 6 months-2 years	420	0.91	0.96	0.048** (0.024)	0.048** (0.023)	412	0.98	0.93	-0.048** (0.02)	-0.049** (0.019)

Indicators	Bihar					Madhya Pradesh				
	N	Mean (Comp-arison)	Mean (Interv-ention)	Mean Diff. (Std. Err.)		N	Mean (Comp-arison)	Mean (Interv-ention)	Mean Diff. (Std. Err.)	
				District Pairs	Village Pairs				District Pairs	Village Pairs
Vaccination	420	0.16	0.19	0.031 (0.036)	0.029 (0.037)	412	0.6	0.7	0.096** (0.047)	0.098** (0.047)
Vitamin A supplements	420	0.06	0.06	-0.005 (0.023)	-0.005 (0.024)	412	0.71	0.69	-0.021 (0.044)	-0.02 (0.047)
Growth monitoring	420	0.44	0.52	0.076 (0.047)	0.072 (0.045)	412	0.96	0.95	-0.009 (0.021)	-0.01 (0.022)
Pre-school education to children aged 3 years and above	420	0.99	1.00	0.014* (0.008)	0.014* (0.008)	412	0.99	0.98	-0.01 (0.012)	-0.01 (0.012)
**AWW Knowledge**										
% AWWs who knew at least half the correct messages and checks to be given to women in last trimester of pregnancy	420	0.26	0.26	-0.008 (0.041)	-0.005 (0.041)	412	0.29	0.25	-0.041 (0.043)	-0.044 (0.043)
% AWWs who knew at least half the correct messages and checks to be given to women who have recently delivered a child	420	0.09	0.09	0.004 (0.026)	0.005 (0.028)	412	0.12	0.06	-0.059** (0.026)	-0.059** (0.026)
% AWWs who knew at least half the correct messages and checks for women with a child aged 6 months	420	0.13	0.16	0.031 (0.032)	0.033 (0.034)	412	0.23	0.16	-0.069* (0.037)	-0.069* (0.039)
% AWWs with knowledge* on Pregnancy care and Birth Preparedness, as per the technical quiz	420	0.77	0.66	-0.107** (0.042)	-0.11*** (0.04)	412	0.64	0.65	0.004 (0.046)	0.01 (0.045)
% AWWs with knowledge on New Born Care in the technical quiz	420	0.33	0.26	-0.065 (0.043)	-0.067 (0.043)	412	0.34	0.38	0.045 (0.047)	0.049 (0.046)

Indicators	Bihar					Madhya Pradesh				
	N	Mean (Comp-arison)	Mean (Interv-ention)	Mean Diff. (Std. Err.)		N	Mean (Comp-arison)	Mean (Interv-ention)	Mean Diff. (Std. Err.)	
				District Pairs	Village Pairs				District Pairs	Village Pairs
% AWWs with knowledge on Family Planning in the technical quiz	420	0.84	0.88	0.039 (0.032)	0.038 (0.034)	412	0.86	0.9	0.04 (0.031)	0.039 (0.033)
% AWWs with knowledge on Breastfeeding in the technical quiz	420	0.85	0.85	0.001 (0.034)	0 (0.033)	412	0.84	0.87	0.031 (0.034)	0.029 (0.035)
% AWWs with knowledge on Complementary feeding in the technical quiz	420	0.99	0.99	0 (0.01)	0 (0.01)	412	0.99	0.97	-0.019 (0.015)	-0.02 (0.016)
% AWWs who correctly answered at least half the questions in the Growth Monitoring quiz	420	0.45	0.45	-0.001 (0.042)	-0.005 (0.042)	412	0.68	0.72	0.042 (0.044)	0.044 (0.044)
