# Supplementary material for: Effects of an mHealth intervention for community health workers on maternal and child nutrition and health service delivery in India: protocol for a quasi-experimental mixed-methods evaluation
Source: BMJ Open. 2019 Mar 27;9(3):e025774. doi: 10.1136/bmjopen-2018-025774 (PMC6475202; doi:10.1136/bmjopen-2018-025774)
Supplement: Supplementary data [file bmjopen-2018-025774supp004.pdf]

Balance in Primary and Secondary Outcome Indicators

Indicators	Bihar					Madhya Pradesh				
	N	Mean (Comp-arison)	Mean (Interv-ention)	Mean Diff. (Std. Err.)		N	Mean (Comp-arison)	Mean (Interv-ention)	Mean Diff. (Std. Err.)	
				District Pairs	Village pairs				District Pairs	Village pairs
**Primary Outcomes**										
% of beneficiaries with 0-6m child who report at least two home visits by Anganwadi Workers (AWW) during their last trimester of pregnancy	810	0.05	0.08	0.026 (0.018)	0.031** (0.015)	757	0.2	0.23	0.036 (0.032)	0.035 (0.028)
% of beneficiaries with 0-6m child who received at least half the messages as per Integrated Child Development Scheme (ICDS) guidelines during their last trimester of pregnancy	810	0.08	0.12	0.038* (0.022)	0.039** (0.019)	757	0.2	0.23	0.037 (0.031)	0.027 (0.027)
% beneficiaries with 0-6m child who received any visit from AWW within 24 hours after birth	810	0.06	0.05	-0.016 (0.017)	-0.018 (0.015)	757	0.07	0.1	0.032 (0.021)	0.04** (0.018)
% beneficiaries with 0-6m child who received at least 2 visits within the first week of birth	810	0.03	0.03	0.002 (0.012)	0.001 (0.01)	757	0.04	0.05	0.003 (0.016)	0.004 (0.014)
% beneficiaries with 0-6m child who received appropriate number of visits as per ICDS guidelines within first six months of birth	810	0.02	0.03	0.014 (0.012)	0.013 (0.011)	757	0.04	0.04	0 (0.016)	0.002 (0.013)
% beneficiaries with 6-12m child who received appropriate number of visits as per ICDS guidelines since their child turned 6 months	444	0.03	0.05	0.023 (0.018)	0.025 (0.018)	465	0.14	0.12	-0.027 (0.031)	-0.03 (0.033)

Indicators	Bihar					Madhya Pradesh				
	N	Mean (Comp-arison)	Mean (Interv-ention)	Mean Diff. (Std. Err.)		N	Mean (Comp-arison)	Mean (Interv-ention)	Mean Diff. (Std. Err.)	
				District Pairs	Village pairs				District Pairs	Village pairs
% of beneficiaries with 6-12m child who received at least half the messages as per ICDS guidelines since their child turned 6 months	444	0	0.01	0.005 (0.008)	0.005 (0.008)	465	0.07	0.05	-0.019 (0.022)	-0.019 (0.022)
% beneficiaries with 12-24m child who received at least 2 visits since their child turned 12 months	431	0.97	0.96	-0.009 (0.018)	-0.011 (0.017)	431	0.94	0.91	-0.035 (0.025)	-0.03 (0.028)
% beneficiaries with child 0-24m who reported that their child was weighed by AWW in the last 3 months	1685	0.16	0.24	0.085*** (0.024)	0.085*** (0.021)	1653	0.54	0.6	0.057** (0.028)	0.057*** (0.022)
% beneficiaries with child 0-24m who reported that AWW discussed their child's weight and nutritional status in the last 3 months	1685	0.11	0.17	0.063*** (0.02)	0.063*** (0.017)	1653	0.37	0.39	0.021 (0.029)	0.021 (0.021)
**Secondary Outcomes**										
**Knowledge about Pregnancy care, Birth preparedness, Health and Nutrition**										
% beneficiaries with 0-6m child who are aware of at least half of the things a pregnant woman should do to take care of herself and baby during pregnancy	810	0.57	0.52	-0.052 (0.04)	-0.047 (0.034)	757	0.59	0.53	-0.06 (0.037)	-0.06** (0.03)
% beneficiaries with 0-6m child who are aware of at least half of the infant danger signs	810	0.04	0.03	-0.016 (0.014)	-0.013 (0.012)	757	0.02	0.06	0.04*** (0.015)	0.041*** (0.013)

Indicators	Bihar					Madhya Pradesh				
	N	Mean (Comparison)	Mean (Intervention)	Mean Diff. (Std. Err.)		N	Mean (Comparison)	Mean (Intervention)	Mean Diff. (Std. Err.)	
				District Pairs	Village pairs				District Pairs	Village pairs
% of beneficiaries with 0-6m child who know how soon an infant should be breastfed after birth of the child	810	0.7	0.72	0.022 (0.033)	0.025 (0.027)	757	0.67	0.72	0.055 (0.034)	0.055* (0.029)
% beneficiaries with 0-6m child who know that infants should not be given water when the infant is less than 6m old	810	0.43	0.35	-0.086** (0.036)	-0.091*** (0.03)	757	0.35	0.37	0.023 (0.036)	0.032 (0.031)
**Services received during pregnancy**										
% currently pregnant women who registered their pregnancy with either Accredited Social Health Activist (ASHA), Auxiliary Nurse Midwife (ANM), AWW, or Govt. Center	1458	0.95	0.97	0.017 (0.011)	0.021** (0.009)	983	0.97	0.99	0.01 (0.009)	0.008 (0.007)
% currently pregnant women who received ANY Antenatal Care (ANC) check-up during their pregnancy	1458	0.83	0.85	0.024 (0.023)	0.029 (0.02)	983	0.93	0.98	0.051*** (0.015)	0.038*** (0.011)
% currently pregnant women who report attending at least one Villahe Health and Nutrition Day (VHND) during their pregnancy	1458	0.47	0.5	0.029 (0.035)	0.022 (0.028)	983	0.65	0.57	-0.089** (0.036)	-0.086*** (0.033)
**Ante-natal Care (ANC)**										
% beneficiaries with 0-24m child who received at least 4 ANC check-ups during pregnancy	1685	0.25	0.2	-0.05** (0.023)	-0.05*** (0.017)	1653	0.52	0.51	-0.003 (0.028)	-0.005 (0.022)
% beneficiaries with 0-24m child who consumed Iron Folic Acid for more than 90 days during pregnancy	1685	0.12	0.12	0.001 (0.017)	0.001 (0.013)	1653	0.54	0.49	-0.054** (0.027)	-0.055** (0.022)

Indicators	Bihar					Madhya Pradesh				
	N	Mean (Comp-arison)	Mean (Interv-ention)	Mean Diff. (Std. Err.)		N	Mean (Comp-arison)	Mean (Interv-ention)	Mean Diff. (Std. Err.)	
				District Pairs	Village pairs				District Pairs	Village pairs
% beneficiaries with 0-24m child who received 2 Tetanus Toxoid (TT) injections during pregnancy	1685	0.84	0.81	-0.033 (0.02)	-0.032** (0.016)	1653	0.81	0.87	0.061*** (0.02)	0.061*** (0.016)
% beneficiaries with 0-24m child who delivered at a health facility	1685	0.73	0.65	-0.079*** (0.028)	-0.079*** (0.021)	1653	0.81	0.81	0.008 (0.022)	0.007 (0.016)
**Beneficiary practices post-delivery**										
% beneficiaries with 0-24m child who breastfed their child within an hour of birth	1685	0.76	0.72	-0.04 (0.025)	-0.04** (0.018)	1653	0.72	0.75	0.024 (0.026)	0.022 (0.019)
% beneficiaries with 0-24m child who had applied nothing to the cord after birth	1685	0.57	0.59	0.025 (0.028)	0.026 (0.021)	1653	0.6	0.6	0.006 (0.027)	0.007 (0.021)
**AWW Home Visits**										
% currently pregnant women who report ANY home visits by AWW during pregnancy	1458	0.23	0.3	0.065** (0.028)	0.057** (0.023)	983	0.39	0.42	0.025 (0.039)	0.012 (0.034)
% currently pregnant women who report that the AWW discussed or checked their Iron Folic Acid intake during a home visit	1458	0.1	0.19	0.091*** (0.024)	0.085*** (0.019)	983	0.36	0.39	0.034 (0.038)	0.024 (0.033)
% currently pregnant women who report that the AWW asked about ANC check-ups, immunization, blood tests, etc. during a home visit	1458	0.13	0.19	0.061** (0.026)	0.047** (0.021)	983	0.31	0.37	0.059* (0.035)	0.053* (0.031)

Indicators	Bihar					Madhya Pradesh				
	N	Mean (Comp-arison)	Mean (Interv-ention)	Mean Diff. (Std. Err.)		N	Mean (Comp-arison)	Mean (Interv-ention)	Mean Diff. (Std. Err.)	
				District Pairs	Village pairs				District Pairs	Village pairs
% beneficiaries with 0-6m child who report that AWW discussed child's birth weight and health status during home visit	810	0.04	0.06	0.021 (0.016)	0.021 (0.014)	757	0.12	0.13	0.013 (0.025)	0.012 (0.021)
% beneficiaries with 6-24m child who reported AWWs discussing child's weight and adequacy of growth during home visit	875	0.03	0.07	0.042*** (0.016)	0.042*** (0.014)	896	0.21	0.14	-0.064** (0.028)	-0.063*** (0.023)
**Family Planning**										
% beneficiaries with 0-24m child who received advice on family planning needs, methods, resources	1685	0.02	0.04	0.023*** (0.008)	0.023*** (0.007)	1653	0.08	0.09	0.004 (0.015)	0.004 (0.011)
% beneficiaries with 0-24m child who is currently using family planning	1685	0.3	0.27	-0.023 (0.025)	-0.023 (0.019)	1653	0.37	0.34	-0.023 (0.025)	-0.023 (0.019)
**Take Home Ration (THR)**										
% currently pregnant beneficiaries who received THR from the AWC during pregnancy	1458	0.53	0.4	-0.131*** (0.034)	-0.127*** (0.027)	983	0.9	0.88	-0.026 (0.022)	-0.023 (0.019)
% beneficiaries with 6-24m child who received THR in the first 6 months after birth of child	875	0.71	0.58	-0.129*** (0.035)	-0.123*** (0.03)	896	0.91	0.92	0.007 (0.019)	0.008 (0.015)
% beneficiaries with 6-24m child who received THR for child > 6m	875	0.5	0.43	-0.068* (0.036)	-0.06** (0.03)	896	0.75	0.77	0.026 (0.031)	0.029 (0.025)
% beneficiaries with 0-24m child who consumed entire THR themselves during pregnancy	1685	0.23	0.19	-0.038 (0.023)	-0.038** (0.018)	1653	0.33	0.39	0.063** (0.026)	0.062*** (0.02)
**Infant and Young Child Feeding (IYCF) - Knowledge and Practices**										

Indicators	Bihar					Madhya Pradesh				
	N	Mean (Comp-arison)	Mean (Interv-ention)	Mean Diff. (Std. Err.)		N	Mean (Comp-arison)	Mean (Interv-ention)	Mean Diff. (Std. Err.)	
				District Pairs	Village pairs				District Pairs	Village pairs
% beneficiaries with 6-12m child who know the right time to start complementary feeding	444	0.95	0.93	-0.021 (0.023)	-0.02 (0.022)	465	0.88	0.9	0.015 (0.029)	0.01 (0.027)
% infants 0-5m of age who received only breastmilk during the previous day	810	0.6	0.51	-0.087*** (0.033)	-0.088*** (0.028)	757	0.46	0.5	0.032 (0.034)	0.038 (0.03)
% infants 6-8m of age who received solid, semi-solid or soft foods during the previous day	266	0.53	0.55	0.016 (0.06)	0.034 (0.088)	264	0.56	0.62	0.051 (0.06)	0.137 (0.094)
% children 6-24m of age who received foods from >= 4 food groups during the previous day	875	0.12	0.15	0.038* (0.023)	0.04** (0.019)	896	0.17	0.22	0.052* (0.027)	0.054** (0.022)
**Growth Monitoring**										
% beneficiaries with 0-6m child who were aware that growth monitoring for children was one of the services at AWCs	810	0.1	0.14	0.039 (0.026)	0.037* (0.021)	757	0.3	0.21	-0.093*** (0.033)	-0.099*** (0.027)
% beneficiaries with 0-24m child who reported that child birth weight as available	1685	0.67	0.58	-0.08*** (0.028)	-0.081*** (0.021)	1653	0.79	0.81	0.02 (0.021)	0.02 (0.015)
% beneficiaries with 0-24m child whose child's birth weight was verified from Mother-Child Protection (MCP)/Health card	1685	0.16	0.15	-0.011 (0.019)	-0.011 (0.014)	1653	0.24	0.29	0.045* (0.024)	0.043** (0.019)
**Diet and Food security**										

Indicators	Bihar					Madhya Pradesh				
	N	Mean (Comp-arison)	Mean (Interv-ention)	Mean Diff. (Std. Err.)		N	Mean (Comp-arison)	Mean (Interv-ention)	Mean Diff. (Std. Err.)	
				District Pairs	Village pairs				District Pairs	Village pairs
% mothers of 0-24m children who have consumed at least five out of ten defined food groups the previous day or night	1685	0.16	0.18	0.015 (0.021)	0.015 (0.016)	1653	0.14	0.16	0.015 (0.021)	0.016 (0.016)
% households which are food secure[1]	1685	0.79	0.7	-0.086*** (0.023)	-0.086*** (0.018)	1653	0.76	0.75	-0.01 (0.023)	-0.011 (0.018)
**Immunization**										
% beneficiaries whose child aged 0-24m ever received any immunizations	1685	0.97	0.97	0 (0.009)	0 (0.007)	1653	0.97	0.98	0.01 (0.008)	0.009 (0.006)
% beneficiaries with 0-24m child who are aware that AWW records details of immunization	1685	0.26	0.3	0.039 (0.025)	0.039** (0.018)	1653	0.32	0.31	-0.016 (0.027)	-0.016 (0.021)
% beneficiaries with 0-24m child who reported that they had MCP cards	1685	0.86	0.86	-0.005 (0.018)	-0.005 (0.014)	1653	0.74	0.91	0.174*** (0.022)	0.175*** (0.017)
% beneficiaries with 0-24m child whose MCP cards were available at home or at Anganwadi Center, and were at least partially filled	1685	0.63	0.65	0.021 (0.025)	0.021 (0.02)	1653	0.54	0.69	0.15*** (0.027)	0.149*** (0.021)
% beneficiaries whose child aged 12-24m is fully immunized	431	0.31	0.35	0.041 (0.045)	0.043 (0.044)	431	0.31	0.4	0.096** (0.044)	0.097** (0.046)
% beneficiaries whose child aged 12-24m is fully immunized (among the subset of beneficiaries with available MCP cards)	293	0.43	0.52	0.083 (0.059)	0.059 (0.082)	268	0.53	0.57	0.069 (0.062)	0.132 (0.096)

[1] Food and Agricultural Organization (FAO) "Food Insecurity Experience Scale Survey Module (FIES-SM) – Household Referenced" (http://www.fao.org/3/a-bl404e.pdf)
