# Supplementary material for: Effects of an mHealth intervention for community health workers on maternal and child nutrition and health service delivery in India: protocol for a quasi-experimental mixed-methods evaluation
Source: BMJ Open. 2019 Mar 27;9(3):e025774. doi: 10.1136/bmjopen-2018-025774 (PMC6475202; doi:10.1136/bmjopen-2018-025774)
Supplement: Author's manuscript [file bmjopen-2018-025774.draft_revisions.pdf]

BMJ Open

BMJ Open is committed to open peer review. As part of this commitment we make the peer review history of every article we publish publicly available.

When an article is published we post the peer reviewers' comments and the authors' responses online. We also post the versions of the paper that were used during peer review. These are the versions that the peer review comments apply to.

The versions of the paper that follow are the versions that were submitted during the peer review process. They are not the versions of record or the final published versions. They should not be cited or distributed as the published version of this manuscript.

BMJ Open is an open access journal and the full, final, typeset and author-corrected version of record of the manuscript is available on our site with no access controls, subscription charges or pay-per-view fees (http://bmjopen.bmj.com).

If you have any questions on BMJ Open's open peer review process please email info.bmjopen@bmj.com

BMJ Open

Effects of an mHealth intervention for community health workers on maternal and child nutrition and health service delivery in India: Study protocol for a quasi-experimental mixed methods evaluation

Journal:	*BMJ Open*
Manuscript ID	bmjopen-2018-025774
Article Type:	Protocol
Date Submitted by the Author:	08-Aug-2018

Complete List of Authors:	Nimmagadda, Sneha; NEERMAN, Center for Causal Research and Impact Evaluation<br>Gopalakrishnan, Lakshmi; NEERMAN, Center for Causal Research and Impact Evaluation<br>Avula, Rasmi; International Food Policy Research Institute<br>Diamond-Smith, Nadia; University of California San Fransisco<br>Fernald, Lia; University of California, Berkeley, School of Public Health<br>Jain, Anoop; University of California, Berkeley, School of Public Health<br>Mani, Sneha; International Food Policy Research Institute<br>Menon, Purnima; International Food Policy Research Institute<br>Nguyen, Phuong Hong; International Food Policy Research Institute<br>Park, Hannah; University of California San Fransisco<br>Patil, Sumeet; NEERMAN, Center for Causal Research and Impact Evaluation<br>Singh, Prakarsh; Institute of Labour Economics (IZA)<br>Walker, Dilys; University of California San Fransisco

Keywords:	mHealth, child health and nutrition, impact evaluation, Quality in health care < HEALTH SERVICES ADMINISTRATION & MANAGEMENT

Note: The following files were submitted by the author for peer review, but cannot be converted to PDF. You must view these files (e.g. movies) online.
Figure 2.tif

SCHOLARONE™ Manuscripts

Effects of an mHealth intervention for community health workers on maternal and child nutrition and health service delivery in India: Study protocol for a quasi-experimental mixed methods evaluation

Authors

Authors	Affiliation	Contact Information
Nimmagadda, Sneha	Network for Engineering, Economics, Research and Management (NEERMAN)	sneha.n@neerman.org
Gopalakrishnan, Lakshmi	Network for Engineering, Economics, Research and Management (NEERMAN)	lakshmi.g@neerman.org
Avula, Rasmi, PhD	International Food Policy Research Institute (IFPRI)	r.avula@cgiar.org
Diamond-Smith, Nadia, PhD	University of California at San Francisco (UCSF)	Nadia.Diamond-Smith@ucsf.edu
Fernald, Lia, PhD	University of California at Berkeley (UCB)	fernald@berkeley.edu
Jain, Anoop	University of California at Berkeley (UCB)	anoopjain@berkeley.edu
Mani, Sneha	International Food Policy Research Institute (IFPRI)	s.mani@cgiar.org
Menon, Purnima, PhD	International Food Policy Research Institute (IFPRI)	p.menon@cgiar.org
Nguyen, Phuong, PhD	International Food Policy Research Institute (IFPRI)	P.H.Nguyen@cgiar.org
Park, Hannah	University of California at San Francisco (UCSF)	hannah.park@ucsf.edu
Patil, Sumeet, PhD	Network for Engineering, Economics, Research and Management (NEERMAN)	srpatil@neerman.org
Singh, Prakarsh, PhD	Institute of Labour Economics (IZA)	prakarsh9@gmail.com
Walker, Dilys, PhD	University of California at San Francisco (UCSF) and University of California at Berkeley (UCB)	dilys.walker@ucsf.edu

Corresponding author: Patil, Sumeet

For peer review only

ABSTRACT

Introduction: Millions of children in India still suffer from poor health and under-nutrition, despite substantial improvement over decades of public health programs. The Anganwadi centers under the Integrated Child Development Scheme (ICDS) provide a range of health and nutrition services to pregnant women, children <6y and their mothers. However, major gaps exist in the ICDS service delivery. The government is currently strengthening the ICDS through an mHealth intervention called Common Application Software (ICDS-CAS), installed on smart phones, with accompanying multi-level data dashboards. This system is intended to be a job aid for frontline workers, supervisors and managers, and aims to ensure better service delivery and supervision, and enable real time monitoring and data-based decision making. However, there is little to no evidence on the effectiveness of such large-scale mHealth interventions integrated with public health programmes in resource constrained settings on the service delivery and subsequent health and nutrition outcomes.

Methods and analysis: This study uses a village-matched controlled design with repeated cross-sectional surveys to evaluate whether ICDS-CAS can enable more timely and appropriate services to pregnant women, children <12 months and their mothers, compared to the standard ICDS programme. The study will recruit approximately 1500 Anganwadi workers and 6000+ mother-child dyads from 400+ matched pairs of villages in Bihar and Madhya Pradesh. The primary outcomes are the proportion of beneficiaries receiving (a) adequate number of home visits and (b) appropriate level of counselling by the Anganwadi workers. Secondary outcomes are related to improvements in other ICDS services, and knowledge and practices of the Anganwadi workers and beneficiaries.

Ethics: Ethical oversight is provided by the Committee for the Protection of Human Subjects at the University of California at Berkeley, and the Suraksha Independent Ethics Committee in India. The results will be published in peer-reviewed journals and analysis data will be made public.

Trial registration number: https://doi.org/10.1186/ISRCTN83902145

For peer review only

STRENGTHS AND LIMITATIONS

- The study can provide important evidence on whether and the extent to which a large-scale *mHealth* intervention can improve the maternal and child health and nutrition service delivery by the world's largest child development program in India.
- The study uses a quasi-experimental design consisting of matched control villages and repeated cross-sectional measurements, which is well suited because of the rapid roll-out of the program and pre-determined programme assignment; both of which precluded the use of more robust cluster-randomized evaluation design.
- Measurement biases may exist because blinding is not possible and primary outcomes are measured subjectively via interview-based recall or observations.
- Higher order impacts may be underestimated as the follow-up period of <12m may be too short for the intervention to stabilize.

For peer review only

INTRODUCTION

Background and Problem Context

Millions of children in India continue to suffer from poor health and under-nutrition, despite decades of government programs aiming to reduce this burden and some impressive gains through these years. In 2015-16, 36% of children under 5 years of age were underweight, 38% were stunted, and 21% were wasted as per the National Family Health Survey (NFHS-4), and these numbers represent only modest improvements over the past decade. Micronutrient deficiencies are widespread, with more than 58% of preschool children suffering from iron deficiency anemia. Infant and neonatal mortality rates also remain high at 41 and 30 per 1000 live births respectively, despite substantive reductions over past decades.[1]

The Integrated Child Development Services (ICDS), launched in 1975, is one of India's national flagship programs to support the health, nutrition, and development needs of children below 6 years of age, and pregnant and lactating women, through a network of Anganwadi Centers (AWCs), each typically serving a population of 800-1000.[2,3] Early observational studies found that the ICDS is associated with better coverage and delivery of services related to nutrition, health care, and pre-school education, and improved maternal and child nutrition.[4–6] Using the NFHS data from 2005-06, Kandpal[7] and Jain[8] found that ICDS is associated with small to modest improvements in child health and nutrition especially among the most vulnerable populations.

However, several reviews and evaluations of the ICDS over the past 18 years have also found persistent gaps including inadequate infrastructure at the AWC, Anganwadi worker (AWW) service delivery issues (e.g., poor quality supplementary food, few home visits, no counselling etc.), human resource issues (e.g., vacancies, increasing range of duties expected of the AWWs, inadequate training of AWWs, limited supervision, etc.), and poor data management (e.g., irregularities in record keeping at AWCs, ineffective monitoring of service delivery, etc.).[3,4,9–11] The most recent NFHS also highlights the gaps in the ICDS service delivery. Only about 59% of the children under 6 years received any service from an AWC, 53% received supplementary food services, and 47% were weighed. Similarly, only 60% of mothers received any AWC services during pregnancy, and only 54% received any service during the breastfeeding phase.[1]

With a goal to improve the functioning of the ICDS, the Government of India launched the ICDS Systems Strengthening and Nutrition Improvement Programme (ISSNIP) in 2012 which focused on infrastructure upgradation and training of AWWs to build their knowledge on health and nutrition topics under the Incremental Learning Approach. At the same time, a pilot-scale mHealth intervention to improve ICDS service delivery was implemented in Bihar between 2012 and 2013. A randomized controlled trial of this intervention found a significant increase in the proportion of beneficiaries receiving visits from frontline workers at different life stages - last trimester of pregnancy (42% vs. 52%), first week after delivery (60% vs. 73%), and complementary feeding stage >5 months after delivery (36% vs. 45%).[12] The intervention also significantly increased the proportion of beneficiaries receiving at least three ANC visits

For peer review only

(29% vs. 50%), the proportion of beneficiaries consuming at least 90 Iron Folic Acid tablets during pregnancy (11% vs. 17%), the proportion of mothers immediately breastfeeding after birth (62% vs. 76%) and the proportion of mothers starting complementary feeding at the right time (32% vs. 41%). Subsequently, the ISSNIP was restructured in 2015 by integrating ICDS in seven states with an at-scale *mHealth* intervention called Common Application Software (ICDS-CAS), installed on smart phones, and with accompanying multi-level data dashboards. This system is intended to be a job aid for frontline workers, supervisors and managers, and aims to ensure better service delivery and supervision, and enable real time monitoring and data-based decision making.

While there is a growing body of evidence on the effectiveness of mHealth interventions, it almost entirely consists of small-scale studies or pilot interventions under well controlled settings, and often of poor research quality. For example, a systematic review examining 17 studies set in low- and middle-income countries found that small scale mHealth interventions, particularly those delivered using SMS, were associated with increased utilization of health care, including uptake of recommended prenatal and postnatal care consultation, skilled birth attendance, and vaccination, but only two of these studies were graded as being at *low risk of bias*.[13] Barnett and Gallegos[14] reviewed nine studies that assessed the impact of using of mobile phones for health and nutrition surveillance, and found that while the available evidence suggests that mobile phones may play an important role in nutrition surveillance by reducing the time required to collect data and by enhancing data quality, the available evidence is of poor methodological quality, generally based on small pilot studies and mainly focuses on feasibility issues. Another recent systematic review of 25 studies found evidence that mobile tools helped community health workers improve the quality of care provided, efficiency of services, and capacity for program monitoring.[15] However, most of these studies were pilots and provided little or no information about the effectiveness of mHealth interventions when integrated with large-scale public health programmes.

This study seeks to address this critical gap in the evidence base in the context of the largest public health and nutrition program in the world, the ICDS, with 1.4 million AWCs serving at the grassroots levels across India. The impact evaluation is conducted in two large states in India, Madhya Pradesh and Bihar, and uses a quasi-experimental, matched-controlled pre-post measurement design. The overall evaluation framework consists of additional components such a process evaluation, a technology evaluation and an economic evaluation.

This evaluation is also timely as India launched the National Nutrition Mission on 8th March 2018 with the goal of reducing malnutrition in a phased manner across entire India and subsumed ISSNIP and ICDS-CAS under it.[16] Therefore, ISSNIP and ICDS-CAS are poised to be scaled up rapidly to reach almost the entire population of India through 1.4 million AWCs by 2020. This scale-up effort will be informed by robust evidence on the effectiveness of the mHealth intervention, as well as on how its implementation can be improved.

The ICDS-CAS Intervention

For peer review only - http://bmjopen.bmj.com/site/about/guidelines.xhtml

Currently, the ICDS-CAS intervention is being implemented at scale in seven states covering over 107,000 AWCs, and through them, a population of 9.8 million registered beneficiaries. The intervention consists of two components as follows.[17]

(1) An android CAS application and smart phones for AWWs and Lady Supervisor: The CAS app was developed on an open source mobile platform (CommCare). The app digitizes and automates ten of the eleven ICDS paper registers maintained by AWWs, enables name-based tracking of beneficiaries, prioritize home visits at critical life stages through home-scheduler, improves record keeping and retrieval of growth or nutrition status of children, helps track immunization, monitors timeliness and quality of different services delivered by AWWs, and includes checklists and videos as job aids. A lady supervisor typically manages a cluster of 10-20 AWCs and the CAS App is expected to help her monitor AWWs remotely, assess quality of service delivery, and serves as a job aid to train AWWs. The app is installed on new smartphones that are provided to the AWWs and supervisors. Both AWWs and supervisors are trained on the use of Apps and how the features help them improve service delivery. Helpdesks at block and district levels for technical support are also established.

(2) A web-enabled dashboard for real-time monitoring by ICDS officials: Data generated at the AWC-level are aggregated and analyzed via web-enabled dashboards for Child Development Project Officers at the project-level (typically an administrative block with 80-100 AWCs), District Programme Officers, a state's ICDS Directorate, and the Ministry of Women and Child Development at the national-level. For example, the monthly progress reports are prepared manually at the AWC-level and then aggregated to the project-level, which requires weeks to be finalized and reviewed, but the CAS App and dashboards will automate and produce these reports in almost real time. The dashboard infographics are expected to help identify bottlenecks at various levels more efficiently, help prioritize local issues, and allow managers to take data-driven actionable decisions.

Figure 1 presents the ICDS-CAS information flow from the AWC through to the MWCD. The logic model in **Figure 2** summarizes how ICDS-CAS is expected to improve service delivery, and ultimately to improve health and nutritional outcomes in mothers and their children. The listed short-term outcomes are those expected to be achieved in the planned evaluation follow-up period of <12 months. The longer-term outcomes related to improved health and nutrition will be measured (except improvements in the cognitive abilities) and analyzed from a learning perspective, but these remain aspirational in the context of this evaluation study.

Evaluation Framework and Research Objectives

The ICDS-CAS evaluation framework consists of four components – an impact evaluation, a process evaluation, an economic evaluation, and a technology evaluation. This paper describes the protocol for the impact evaluation in detail to guide the final analysis plan. The other three components are summarized in **Supplementary Figure 1** without binding details because their objectives, scope and methods can change as per the evolving learning needs of policy makers. We intend to analyze and publish the impact evaluation and process evaluation as standalone research articles in peer reviewed journals. The analysis and publication plan for the other components is yet to be determined.

For peer review only

Corresponding to the short term strategic objective of ICDS-CAS to improve quality and quantity of AWW and beneficiary interactions, the main research questions the impact evaluation seeks to answer are:

1. Does ICDS-CAS improve the timeliness or frequency of home visits by the AWW to pregnant women, infants and their mothers?
2. Does ICDS-CAS improve extent or level of counselling from AWWs to pregnant women and mothers of infants?

METHODS AND ANALYSIS

Study Setting

The ICDS-CAS program is being implemented in 57 districts from seven ISSNIP states in India where the burden of under-nutrition is highest. This evaluation is restricted to two states, Madhya Pradesh (MP) and Bihar, which were selected because of the possibility of selecting an ISSNIP district as a control, willingness of the states to support the evaluation, and the suggestions by the Ministry of Women and Child Development and the funding agency. **Supplementary Table 1** presents key health and nutrition related indicators for rural villages of India, MP, and Bihar. Both states have a high burden of under-five mortality (69 per 1,000 live births in MP, and 60 in Bihar), stunting (43.6% of children aged 0-5 years in MP, and 49.3% in Bihar), and anemia (>55% of children and pregnant women in both states). The antenatal- and delivery-related indicators are, in general, worse in Bihar, whereas MP has relatively poor demographic, water-sanitation, education, and mortality related indicators– 8.3% of mothers in MP and 3% in Bihar had full antenatal care; 79.5% of households in MP and 98.2% in Bihar had an improved drinking-water source; and 50.2% of children aged 12-23 months in MP and 61.9% in Bihar were fully immunised.

Overview of the Identification Strategy

The attributable effects of ICDS-CAS will be identified using a quasi-experimental matched, controlled design with repeated cross-sectional pre- and post-intervention measurements. This identification strategy is grounded in the Neyman–Rubin potential outcomes model where a matched cohort design can yield unbiased estimates of the causal effects under a strong assumption that all confounders are measured and balanced between the intervention and comparison groups.[18–20] We use a 1:1 nearest neighbour propensity score matching (PSM) method to identify pairs of intervention and comparison villages.[21–25] We plan to identify the effects of ICDS-CAS by comparing post-intervention outcome indicators between the matched groups, while controlling for any pre-intervention or baseline differences in the outcomes averaged at the village level and adjusting for the matched paired design.

Sample Design and Power Calculations

For peer review only

The initial sample size was determined as 400 villages in each arm with one AWW and on average three mother-child dyads in each village to measure a relative effect of 15% in a standardized counterfactual outcome [Normal(0,1)], with a significance level of 0.05, power of 80%, and Intra-Cluster Correlation (ICC) of 0.15. The actual baseline survey sample consisted of 852 villages to account for refusals and loss to follow-up.

After the baseline survey was conducted in June-August 2017 and completely separate from our efforts to design the evaluation, the Indian government decided to include ICDS-CAS in the National Nutrition Mission. Consequently, the original evaluation objectives are being revised to estimate the effects of ICDS-CAS separately for MP and Bihar to draw deeper insights into the heterogeneity of impacts. In case of such an eventuality, the evaluation will need to be powered to detect smaller magnitudes of effects on a few process-related secondary outcomes. If needed, we plan to increase the sample power by following the same panel of villages but recruiting almost twice as many participants in each village – up to two AWWs and up to eight mother-child dyads. Assuming 200 pairs of villages and 1200 mothers per arm in each state, the sample will have 80% power at a significance level of 0.05 to detect absolute difference of 5-9 %-points from the counterfactual levels between 10%-50% with ICC between 0.15-0.30. With 400 AWWs per arm in each state, the sample will have 80% power to detect effects of 8-12 %-points for AWW level outcomes from the counterfactual levels of 10%-40% with ICC between 0.25-0.45.

Sampling and Recruitment of Study Participants

Figure 3 summarizes the sampling and recruitment of study participants for the baseline survey. First, we sampled intervention districts and selected geographically and administratively matched comparison districts. Then, we randomly sampled intervention villages in two steps of sampling the blocks and then the villages. Finally, we pair-matched intervention villages with villages from the comparison districts and selected the best matched 426 pairs as discussed next.

Sampling of Intervention and Comparison Districts: We randomly sampled three pairs of geographically and administratively matched intervention and comparison districts which shared a boundary and belonged to the same ICDS division to control for division-level confounders related to ICDS administration and management as well as cultural, social, environmental, and economic factors at the micro-region scale. Within each state, we first sample three invention districts, and the corresponding control districts were selected *de facto* if only one eligible district was available within the division, or randomly, if multiple comparison districts were available. In MP, the sample purposively included one pair of *tribal-only* districts from an equity-focused evaluation perspective. **Figure 4** depicts the states and districts included in the evaluation.

Selecting Matched Pairs of Treatment and Control Villages: From each selected district, we randomly sampled two blocks in MP and three blocks in Bihar. Next, we randomly sampled 345 villages from six intervention blocks in MP and 315 villages from nine ICDS-CAS blocks in Bihar. In both states, the sample frame was restricted to villages with population of 500 or more to increase the possibility that the villages are sufficiently large to be served by dedicated AWCs. Next, we matched the sampled treatment

For peer review only - http://bmjopen.bmj.com/site/about/guidelines.xhtml

villages with villages from the paired comparison district using a 1:1 nearest neighbour PSM method using variables from Census 2011[1].[20–23]

Table 1 summarizes the matching performance in terms of reduction in the sum of standardized mean bias after matching. The standardized mean bias was reduced by 61-82% after matching across the six district pairs. The Log Reduction (LR) test statistic before matching was statistically significant (*p*-value <0.001), but became insignificant after matching (*p*-value >0.75), suggesting that both groups are statistically similar on average after the matching procedure. We then selected the best matched village pairs– in terms of the difference in the propensity scores of the matched pairs –35 pairs of villages for each intervention block in MP and 24 pairs of villages for each intervention block in Bihar. Overall, we selected 216 matched pairs (432 villages) in Bihar and 210 matched pairs (420 villages) in MP.

Table 1. Comparison of Matching Performance in Reducing Bias

	**Madhya Pradesh**			**Bihar**		
	**Ujjain-Dewas**	**Barwani-Alirajpur**	**Katni-Jabalpur**	**Samastipur-Darbhanga**	**Lakhisarai-Muzaffarpur**	**Sitamarhi-Jamui**
Standardized mean bias - before matching	16.6	27.5	40.3	27	35.4	40.8
Standardized mean bias - after matching	6.5	8	9.9	8.3	6.6	9.7
% reduction in mean bias	61%	71%	75%	69%	81%	76%
p-value of LR test - Before matching	0.000	0.000	0.000	0.000	0.000	0.000
p-value of LR test - After matching	0.929	0.929	0.905	0.788	0.997	0.490
Mean difference in propensity score	0.002	0.072	0.131	0.004	0.046	0.181

Recruitment of Study Participants: In the already conducted baseline survey, we randomly sampled one AWC if more than one AWC existed in a selected village. Using the list of beneficiaries available at the

[1]*Census 2011 variables used for matching: Distance between village and block headquarters (kilometers); Population; % of SC/ST households; % of villages served by public transport; % of villages connected to a major road; % of villages with a public ration shop; % of villages with a post office; % of villages with a bank; Average proportion of households in a village with a bank account; % of villages with an agricultural society; % of villages with a self-help group; Average proportion of households in a village serviced by closed drainage system; Average proportion of households in a village with improved source of drinking water; Average proportion of households in a village with improved sanitation facility; Average proportion of households in a village using electricity as the main source of light; Average proportion of households in a village with a pucca house; Average household asset index for the village*

For peer review only

selected AWC as a sample frame, we randomly sampled four mother-child dyads after stratification by life-stages: (1) mother of child aged <3 months, (2) mother of child aged 3 to <6 months, (3) mother of child aged 6 to <12 months, and (4) mother of child aged 12 to <24 months. Additionally, we randomly sampled up to five pregnant women or mothers with a child <3 months for more in-depth analysis of ICDS services during pregnancy and child birth. To measure AWC-level outcomes, we conduct a survey of the selected AWW as well.

The endline will be a repeated cross-sectional sample where pregnant women and mothers with children<12 months will be recruited using the list of beneficiaries available at the AWC at that time. We do not plan to recruit children older than 12 months at the endline because the ICDS-CAS intervention would be active for <12 months and we can realistically measure the changes only among children who were born after the ICDS-CAS implementation started.

All survey participants are/will be recruited for the study after being administered informed consent as per the IRB-approved protocol. Additional assent is taken just before taking anthropometric measurements (height and weight) of the children.

Patient and Public Involvement

This research was done without patient or public involvement in conceptualization, design, implementation, analysis, manuscript development or dissemination.

Primary Outcomes

The primary outcomes to assess the effectiveness of ICDS-CAS compared to the standard ISSNIP and ICDS are:

1. The proportion of pregnant women and mothers of children <12 months who received adequate number of home visits by the AWW in the past three months; and

2. The proportion of pregnant women and mothers of children <12 months who received appropriate extent or level of counselling from the AWW during their interactions (at home, at AWCs, or in other settings) in the past three months.

Secondary Outcomes

Several outputs and outcomes according to the logic model presented in **Figure 2** are secondary outcomes in this evaluation study as discussed before. These include outcomes related to supervisory and capacity building support to AWWs, infrastructure and supplies at the AWC, AWW level outcomes (motivation, satisfaction, knowledge, time allocation for services and record keeping, time allocation for service delivery, number of beneficiaries served), and additional ICDS services that can be improved by ICDS-CAS but also critically dependent on other external factors (growth monitoring of children, provision of IFA and supplemental nutrition, immunization tracking, referrals, etc.). We will also measure higher order but distal or aspirational outcomes related to knowledge, practices, health and nutrition at the beneficiary level.

For peer review only

Outcome Measurements

All beneficiary- and AWW-level outcomes will be measured through structured interviews, with verification of registers and documents wherever possible. The household instruments were developed using standard questions from the WHO and Demographic and Health Survey frameworks, and capture information about the demographic and socio-economic characteristics of the household, birth history and family planning, pregnancy care, awareness and utilization of AWC services at different life-stages, IYCF practices, immunization, knowledge on health and nutrition, and child health and nutritional outcomes (child growth).The AWW instrument captures information about coverage of beneficiaries, service delivery, supervisory support, work incentives and motivation, training received, time allocation, knowledge, and infrastructure and supplies at the AWC. The interviews are administered in *Hindi* on android tablet-based SurveyCTO™ platform.

Analysis Plan

The effects of the ICDS-CAS intervention will be estimated as,

$$Y_{ij,t=1} = \beta_0 + \beta_1 . T_j + Pair\ ID_K + \bar{Y}_{j,t=0} + \varepsilon$$

where,

$Y_{ij,t=1}$ = an outcome of interest for beneficiary *i* in village *j* at endline (*t*=1);

T_j = indicator variable which is 1 for intervention and 0 for comparison villages;

$Pair\ ID_K$ = Fixed effects to account for *k* pairs of matched villages;

$\bar{Y}_{j,t=0}$ = average pre-intervention or baseline (t = 0) level of the outcome Y in the village;

ε = the error term of the model; and

β_1 = the effect of the intervention on outcome Y.

We will adjust for the pre-intervention average level of the outcome in a village ($\bar{Y}_{j,t=0}$) to control for, *at least*, the measured or unmeasured *time invariant* village- and higher-level confounders. To the extent the endline questionnaires or sampling are different from those in the baseline, the construction of the outcome indicators in the baseline and endline may differ slightly, but such differences (if any) will not affect the interpretation of impact parameters estimated using the above model specification. Additionally, as a consistency check, we will estimate adjusted treatment effects that control for any observed imbalance in important baseline covariates. We may additionally control for covariates that can help increase the precision of impact estimates. We do not plan to correct the *p*-values for multiple comparisons because we only test a limited number of indicators for the primary outcomes. We also plan to estimate the effects by adjusting for pairing only at the district level and not at the village-level if accounting for village matching results in substantial sample loss.

Baseline Balance

For peer review only

Supplementary Table 2 and Supplementary Table 3 present the baseline balance at AWC, household and individual beneficiary levels. The balance is assessed in terms of group mean difference after adjusting for the pairing of villages. As a robustness check, the group mean differences by adjusting only for the district pairing (and not the village pairs) are also presented. Overall, practically-perfect balance in terms of exogenous variables such as household or individual characteristics is achieved, but there are a few differences in the AWC and AWW level characteristics and service delivery as reported by the AWWs. Almost all indicators related to home visits and growth monitoring were balanced as the magnitude of the group differences are of little practical significance, except for the growth monitoring related outcomes in Bihar. A few secondary outcomes were meaningfully different as well. However, such differences are expected when more than 100 covariates and outcome indicators are tested for balance. We also do not see a discernible pattern where control or intervention groups are consistently better or worse off than the other group. Considering the preponderance of highly similar distribution of variables, we infer that the matching resulted in exchangeable or balanced groups, and any residual confounding at the community level can be removed by controlling for the baseline outcomes.

DISCUSSION

The evaluation will provide evidence on whether and to what extent ICDS-CAS *mHealth* can improve health and nutrition service delivery beyond what is feasible with traditional non-technology based approaches under the ISSNIP. Additionally, the analysis of a range of lower order outputs and outcomes can help us identify the pathways through which ICDS-CAS has worked, or the critical failure points.

The study faces a few limitations in identifying unbiased estimates of the programme effects due to the nature of the intervention and constraints on the study design. First, confounding or selection bias cannot be theoretically ruled out in an observational study such as ours. While the matching procedure appears to be successful, it may not have removed all residual and unobserved confounding. Measurement biases including the Hawthorne effect[26] are possible because the outcomes are measured through interview recalls and observations. The external validity of the findings can be questioned because of the purposive sampling of states, pairing of districts, and the PSM matching of villages does not result in a statistically representative sample of ICDS-CAS programme area. Finally, as is the case with most large-scale programmes, ICDS-CAS implementation may be delayed and the planned follow-up period may not be adequate for the impacts to materialise.

While these limitations are common in observational studies, the study team has tried to minimize the risk to validity of the findings by reducing the observed pre-intervention imbalance using a large set of variables from Census 2011for matching, controlling for *at least* the cluster level time-invariant confounders by using a repeated cross sectional design, measuring the primary outcomes at the beneficiary level who will be blinded to their intervention status in the study, measuring a large set of indicators as per the logic model to test whether ICDS-CAS is working through hypothesized pathways, and delaying the endline survey as much as possible before the intervention must be implemented in the control districts. The evaluation framework also includes other components which can assess the intervention using mixed methods approaches, and can help build confidence on the study findings.

For peer review only

Overall, this study will contribute to the evidence base on whether *mHealth* interventions can improve community health workers' efficiency and effectiveness. This is also a highly policy-relevant evaluation which can inform scale up of the intervention to potentially cover the entire country by 2020.

ETHICS AND DISSEMINATION

Study protocols have been reviewed and approved by institutional review boards at the University of California, Berkeley (Ref. No. 2016-08-9092), and the India-based Suraksha Independent Ethics Committee (Protocol No. 2016-08-9092). The trial is registered with The ISRCTN registry (https://doi.org/10.1186/ISRCTN83902145). The results will be published in peer reviewed journals and presented in conferences and dissemination meetings.

DATA SHARING STATEMENT

Deidentified data will be made publicly available after publication of the impact evaluation manuscript as per the funding and publication agreements with Bill and Melinda Gates foundation.

AUTHORS' CONTRIBUTIONS

DW and LF are the principal investigators on the grant from Bill and Melinda Gates Foundation. ND-S, LF, PM, HP, SP, PS, DW contributed to the study design and lead different sub-components of the study, all authors were involved in protocol development and finalising instruments. SN and SP led the sampling. Data collection was mainly managed by SN, LG, RA, SM. SN, LG, SP developed the first draft of the manuscript. All authors reviewed, revised and approved the final manuscript.

FUNDING STATEMENT

This study is funded by Grant No. OPP1158231 from Bill and Melinda Gates Foundation to the University of California, San Francisco and University of California, Berkeley.

COMPETING INTERESTS STATEMENT

None declared.

For peer review only

1 International Institute for Population Sciences (IIPS) and ICF. National Family Health Survey (NFHS-4), 2015-16: India. Mumbai: : IIPS 2017. http://rchiips.org/NFHS/NFHS-4Reports/India.pdf (accessed 7 Mar 2018).

2 Rao N, Kaul V. India's integrated child development services scheme: challenges for scaling up. *Child Care Health Dev* 2018;**44**:31–40. doi:10.1111/cch.12531

3 Gragnolati M, Bredenkamp C, Gupta MD, *et al.* ICDS and Persistent Undernutrition: Strategies to Enhance the Impact. *Econ Polit Wkly* 2006;**41**:1193–201.

4 Kapil U, Pradhan R. Integrated Child Development Services scheme (ICDS) and its impact on nutritional status of children in India and recent initiatives. *Indian J Public Health* 1999;**43**:21.

5 Agarwal KN, Agarwal DK, Agarwal A, *et al.* Impact of the integrated child development services (ICDS) on maternal nutrition and birth weight in rural Varanasi. *Indian Pediatr* 2000;**37**:1321–7.

6 Tandon BN. Nutritional interventions through primary health care: impact of the ICDS projects in India. *Bull World Health Organ* 1989;**67**:77–80.

7 Kandpal E. Beyond Average Treatment Effects: Distribution of Child Nutrition Outcomes and Program Placement in India's ICDS. *World Dev* 2011;**39**:1410–21. doi:10.1016/j.worlddev.2010.12.013

8 India IF. Has the ICDS helped reduce stunting in India? Ideas India. http://www.ideasforindia.in/topics/human-development/has-the-icds-helped-reduce-stunting-in-india.html (accessed 6 Apr 2018).

9 Adhikari S, Bredenkamp C. Moving Towards An Outcomes-Oriented Approach to Nutrition Program Monitoring: The India ICDS Program. 2009.

10 Planning Commission Programme Evaluation Organisation. Evaluation Report on Integrated Child Development Services Vol.I. New Delhi: : Planning Commission, Government of India 2011. http://planningcommission.nic.in/reports/peoreport/peoevalu/peo_icds_v1.pdf (accessed 7 Mar 2018).

11 NITI Aayog Programme Evaluation Organisation. A Quick Evaluation Study of Anganwadis under ICDS. New Delhi: : NITI Aayog 2015. http://niti.gov.in/writereaddata/files/document_publication/report-awc.pdf (accessed 23 Apr 2018).

12 Evaluation of the Information and Communication Technology (ICT) Continuum of Care Services (CCS) Intervention in Bihar. Math. Policy Res. https://www.mathematica-mpr.com/our-publications-and-findings/publications/evaluation-of-the-information-and-communication-technology-ict-continuum-of-care-services-ccs (accessed 23 Mar 2018).

13 Lee SH, Nurmatov UB, Nwaru BI, *et al.* Effectiveness of mHealth interventions for maternal, newborn and child health in low– and middle–income countries: Systematic review and meta–analysis. *J Glob Health* 2016;**6**. doi:10.7189/jogh.06.010401

For peer review only

14 Barnett I, Gallegos JV. Using Mobile Phones for Nutrition Surveillance: A Review of Evidence. IDS 2013. https://opendocs.ids.ac.uk/opendocs/handle/123456789/2602 (accessed 11 Jun 2018).

15 Braun R, Catalani C, Wimbush J, *et al.* Community Health Workers and Mobile Technology: A Systematic Review of the Literature. *PLOS ONE* 2013;**8**:e65772. doi:10.1371/journal.pone.0065772

16 PM launches National Nutrition Mission, and pan India expansion of Beti Bachao Beti Padhao, at Jhunjhunu in Rajasthan. http://pib.nic.in/newsite/PrintRelease.aspx?relid=177166 (accessed 23 Mar 2018).

17 CAS Project Management Unit. Rollout of ICT-RTM Core Deck. 2018.

18 Rubin DB. Causal Inference Using Potential Outcomes. *J Am Stat Assoc* 2005;**100**:322–31. doi:10.1198/016214504000001880

19 Rubin DB. Estimating Causal Effects of Treatments in Randomized and Nonrandomized Studies. *J Educ Psychol* 1974.

20 Arnold BF, Khush RS, Ramaswamy P, *et al.* Causal inference methods to study nonrandomized, preexisting development interventions. *Proc Natl Acad Sci* 2010;**107**:22605–10. doi:10.1073/pnas.1008944107

21 Rubin D, R. Rosenbaum P. Constructing a Control Group Using Multivariate Matched Sampling Methods That Incorporate the Propensity Score. *Am Stat* 1985;**39**. doi:10.1080/00031305.1985.10479383

22 Stuart EA. Matching methods for causal inference: A review and a look forward. *Stat Sci Rev J Inst Math Stat* 2010;**25**:1–21. doi:10.1214/09-STS313

23 Caliendo Marco, Kopeinig Sabine. Some practical guidance for the implementation of propensity score matching. *J Econ Surv* 2008;**22**:31–72. doi:10.1111/j.1467-6419.2007.00527.x

24 Rosenbaum PR, Rubin DB. The central role of the propensity score in observational studies for causal effects. *Biometrika* 1983;**70**:41–55. doi:10.1093/biomet/70.1.41

25 Brady H, Collier D, S. Sekhon J. The Neyman-Rubin Model of Causal Inference and Estimation Via Matching Methods. *Oxf Handb Polit Methodol* Published Online First: 1 January 2008. doi:10.1093/oxfordhb/9780199286546.003.0011

26 Fernald DH, Coombs L, DeAlleaume L, *et al.* An Assessment of the Hawthorne Effect in Practice-based Research. *J Am Board Fam Med* 2012;**25**:83–6. doi:10.3122/jabfm.2012.01.110019

For peer review only

FIGURE LEGENDS

Figure 1. ICDS-CAS information flow from the Anganwadi Center to the Ministry of Women and Child Development. Solid lines correspond to interactions. Dotted lines correspond to data flow. ICDS-CAS, Integrated Child Development Services-Common Application Software; AWW, Anganwadi Worker; CDPO, Child Development Project Officer; MWCD, Ministry of Women and Child Development.

Figure 2. Logic Model of ICDS-CAS and Measurement of Outcomes. MoH, Ministry of Health; ICDS-CAS, Integrated Child Development Services-Common Application Software; AWW, Anganwadi Worker; CDPO, Child Development Project Officer; DPO, District Programme Officer; LS, Lady Supervisor.

Figure 3. Sampling of Study Participants from Madhya Pradesh and Bihar. ISSNIP, ICDS Systems Strengthening and Nutrition Improvement Programme; ICDS, Integrated Child Development Services; CAS, Common Application Software; AWC, Anganwadi Center; AWW, Anganwadi Worker.
- *Source*: Women and Child Development Department, Government of Madhya Pradesh (MIS) http://mpwcdmis.gov.in/(S(ee1m5bxnuzmixun00bsctvfj))/DataEntryAwc.aspx (Accessed 8 June 2018)
& - *Source*: Integrated Child Development Services, Government of Bihar http://www.icdsbih.gov.in/AnganwadiCenters.aspx?GL=16 (Accessed 8 June 2018)
***** - *Source*: Programme documentation from implementing agencies

Figure 4. Sampled Intervention and Comparison Districts. Green areas indicate intervention districts. Blue areas indicate comparison districts.

SUPPLEMENTARY FIGURE LEGEND

Supplementary Figure 1. Components of the ICDS-CAS Evaluation. ICDS-CAS, Integrated Child Development Services-Common Application Software; AWW, Anganwadi Worker; LS, Lady Supervisor; VHND, Village Health and Nutrition Day.

For peer review only

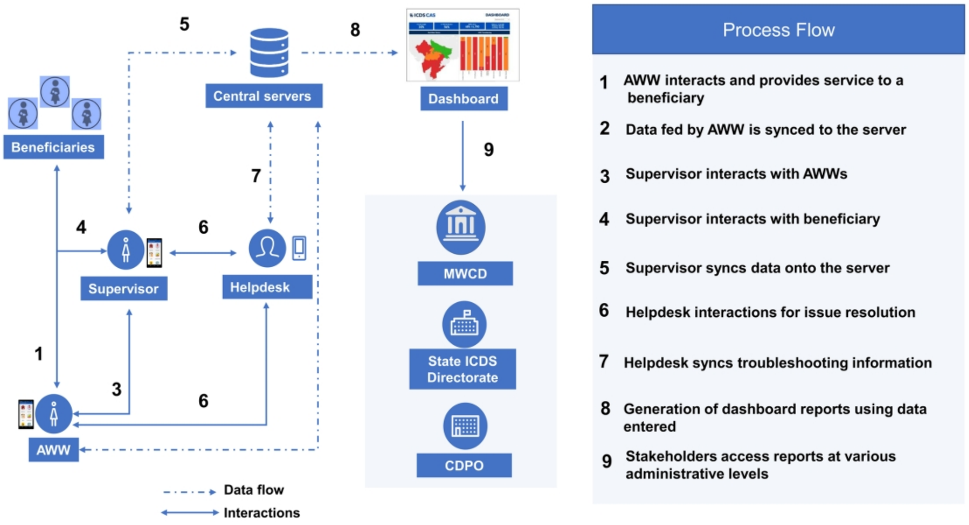

Figure 1. ICDS-CAS information flow from the Anganwadi Center to the Ministry of Women and Child Development.

190x107mm (300 x 300 DPI)

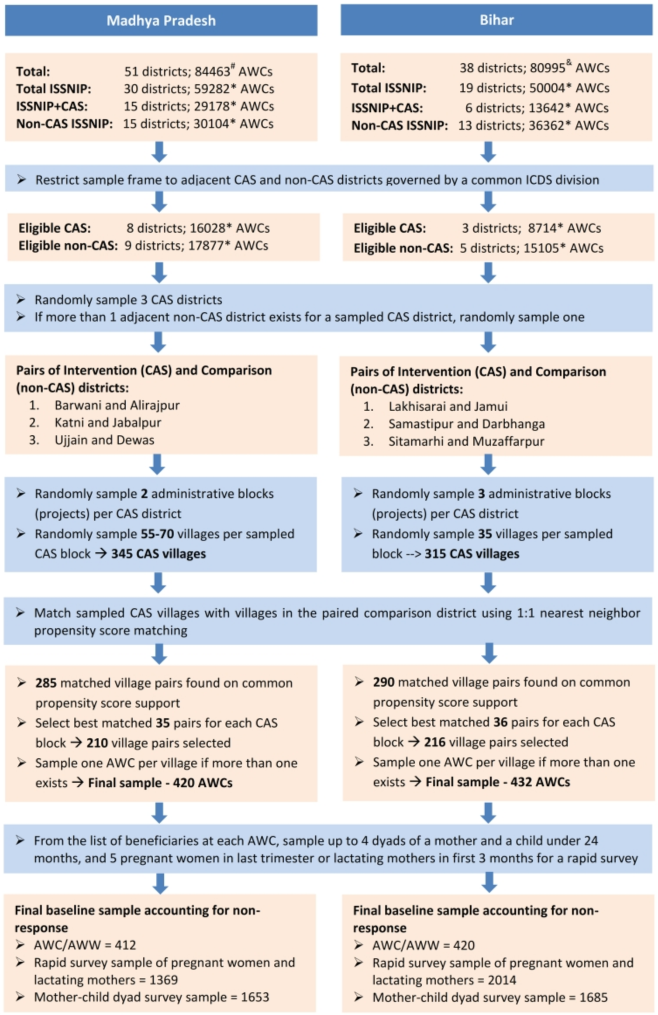

Figure 3. Sampling of Study Participants from Madhya Pradesh and Bihar.

297x420mm (300 x 300 DPI)

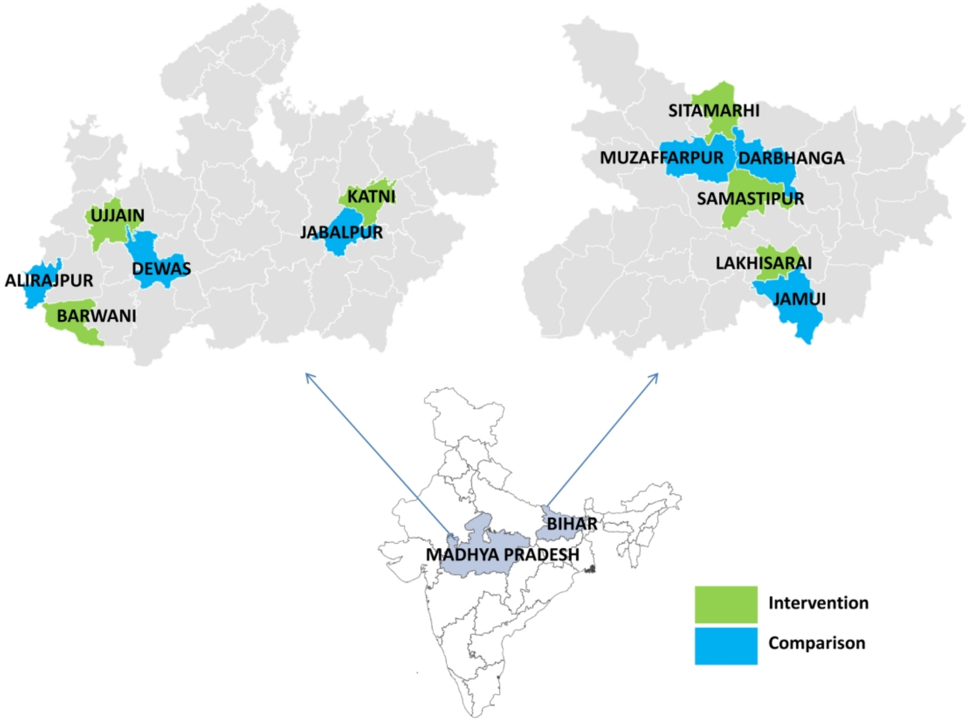

Figure 4. Sampled Intervention and Comparison Districts.

190x142mm (300 x 300 DPI)

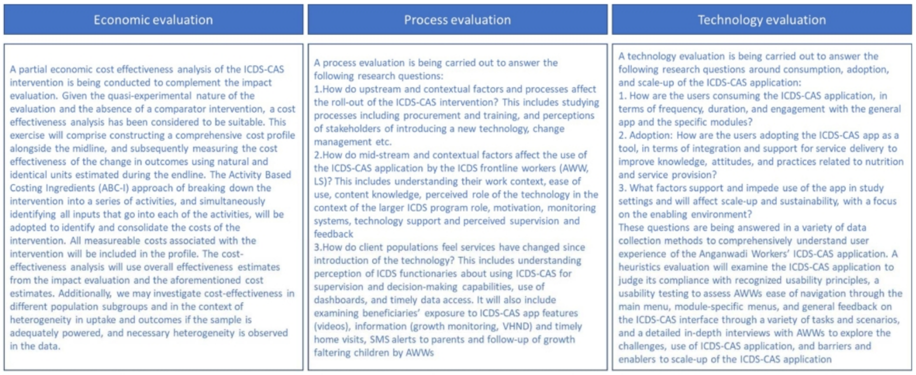

215x166mm (300 x 300 DPI)

Population, Health and Nutrition Related Indicators from National Family Health Survey (NFHS): India, Madhya Pradesh and Bihar, 2015-16

NFHS Indicators	India	Madhya Pradesh	Bihar
Sex ratio of the total population (females per 1,000 males)	1009	955	1075
Children under age 5 years whose birth was registered (%)	76.1	78.4	60.3
Households with electricity (%)	83.2	86.4	54.1
Households using clean fuel for cooking (%)	43.8	29.6	17.8
Households with an improved drinking-water source (%)	89.3	79.5	98.2
Households using improved sanitation facility (%)	36.7	19.4	20.7
Population (female) age 6 years and above who ever attended school (%)	68.8	64.0	56.9
Women with 10 or more years of schooling (%)	27.3	14.1	19.5
Women age 15-19 years who were ever pregnant (%)	9.2	8.6	12.8
Under-five mortality rate (U5MR)	56	69	60
Married women use of family planning methods (%)	51.7	51.3	22.6
Health worker ever talked to female non-users about family planning (%)	17.2	19.3	11.6
Mothers who consumed iron folic acid for 100+ days during pregnancy (%)	25.9	20.5	9.4
Mothers who had full antenatal care (%)	16.7	8.3	3.0
Institutional births (%)	75.1	76.4	62.7
Institutional births in public facility (%)	54.4	70.3	48.2
Children age 12-23 months fully immunized (%)	61.3	50.2	61.9
Children under age 6 months exclusively breastfed (%)	56.0	59.6	54.2
Total children age 6-23 months receiving an adequate diet (%)	8.8	6.0	7.4
Children under 5 years who are stunted (height-for-age) (%)	41.2	43.6	49.3
Children under 5 years who are wasted (weight-for-height) (%)	21.5	27.1	20.8
Children under 5 years who are underweight (weight-for-age) (%)	38.3	45.0	44.6
Women whose Body Mass Index (BMI) is below normal (<18.5 kg/m2) (%)	26.7	31.8	31.8
Children age 6-59 months who are anaemic (<11 g/dl) (%)	59.4	69.9	64.0
Pregnant women age 15-49 years who are anaemic (<11 g/dl) (%)	52.1	56.4	58.0

Source: NFHS-4 fact sheets for India, Madhya Pradesh and Bihar (http://rchiips.org/NFHS/factsheet_NFHS-4.shtml)

For peer review only - http://bmjopen.bmj.com/site/about/guidelines.xhtml

Balance in Covariates at Anganwadi, Household and Individual Beneficiary levels

Indicators	Bihar					Madhya Pradesh				
	N	Mean (Comp-arison)	Mean (Interv-ention)	Mean Diff. (Std. Err.)		N	Mean (Comp-arison)	Mean (Interv-ention)	Mean Diff. (Std. Err.)	
				District Pairs	Village Pairs				District Pairs	Village Pairs
**Respondent Characteristics**										
Average age in completed years	3143	24.31	24.90	0.587*** (0.18)	0.583*** (0.126)	2636	24.18	24.12	-0.065 (0.151)	-0.074 (0.118)
Average education level in years	3143	3.96	3.30	-0.663*** (0.231)	-0.695*** (0.17)	2636	4.55	4.92	0.353* (0.198)	0.35** (0.153)
% of respondents who work to earn income	3143	0.11	0.12	0.014 (0.015)	0.017 (0.011)	2636	0.45	0.44	-0.017 (0.02)	-0.018 (0.014)
% of respondents who have an Aadhaar card	3143	0.97	0.96	-0.015** (0.006)	-0.016*** (0.005)	2636	0.94	0.94	-0.002 (0.01)	-0.004 (0.008)
% of respondents who own a bank account	3143	0.77	0.80	0.029* (0.017)	0.026** (0.013)	2636	0.78	0.81	0.026 (0.019)	0.024* (0.014)
% of respondents who are currently married	3143	1.00	1.00	-0.001 (0.002)	-0.001 (0.002)	2636	0.99	0.99	0 (0.003)	0.001 (0.002)
% of respondents who were residing in their permanent marital home at the time of survey	3143	0.88	0.89	0.012 (0.013)	0.012 (0.009)	2636	0.92	0.9	-0.019 (0.012)	-0.017* (0.009)
% of respondents who were residing in their permanent maternal home at the time of survey	3143	0.02	0.01	-0.011** (0.005)	-0.011*** (0.004)	2636	0.01	0.01	0.002 (0.004)	0.002 (0.003)
**Household Socio-economics**										
% of households which belong to Scheduled Castes or Scheduled Tribe categories	3143	0.35	0.38	0.03 (0.03)	0.031 (0.023)	2636	0.56	0.5	-0.061** (0.028)	-0.06*** (0.021)

For peer review only

Indicators	Bihar					Madhya Pradesh				
	N	Mean (Comp-arison)	Mean (Interv-ention)	Mean Diff. (Std. Err.)		N	Mean (Comp-arison)	Mean (Interv-ention)	Mean Diff. (Std. Err.)	
				District Pairs	Village Pairs				District Pairs	Village Pairs
% of households which belong to Below-Poverty-Line category	3143	**0.58**	**0.62**	0.039* (0.022)	0.043** (0.017)	2636	0.63	0.66	0.024 (0.022)	0.023 (0.017)
% of households with pucca construction	3143	0.38	0.37	-0.007 (0.024)	-0.009 (0.017)	2636	0.26	0.3	0.036 (0.024)	0.033* (0.018)
% of households with fewer than two rooms for sleeping	3143	**0.34**	**0.41**	0.068*** (0.022)	0.067*** (0.016)	2636	0.39	0.41	0.017 (0.023)	0.022 (0.017)
% of households that own agricultural land	3143	0.47	0.42	-0.051** (0.026)	-0.055*** (0.019)	2636	0.76	0.74	-0.021 (0.022)	-0.019 (0.017)
% of households that mainly use improved fuels for cooking	3143	0.17	0.15	-0.024 (0.017)	-0.024* (0.013)	2636	0.1	0.14	0.033** (0.016)	0.032*** (0.011)
% of households with improved drinking water source on premises	3143	**0.61**	**0.55**	-0.053** (0.025)	-0.055*** (0.02)	2636	0.14	0.14	0.001 (0.018)	0.004 (0.013)
% of households with a functional toilet	3143	**0.24**	**0.39**	0.156*** (0.025)	0.152*** (0.019)	2636	**0.52**	**0.46**	-0.061** (0.029)	-0.063*** (0.022)
% of households with any member practicing open defecation	3143	**0.81**	**0.70**	-0.107*** (0.023)	-0.105*** (0.018)	2636	**0.62**	**0.71**	0.086*** (0.027)	0.086*** (0.02)
% of households with piped water	3143	0.01	0.01	0.004 (0.005)	0.004 (0.004)	2636	0.08	0.07	-0.006 (0.017)	-0.006 (0.013)
% of households with a handwashing facility	3143	0.69	0.66	-0.027 (0.029)	-0.032 (0.021)	2636	0.62	0.65	0.025 (0.03)	0.025 (0.022)
**Anganwadi Center (AWC) Coverage**										
Average population in catchment area	419	1111.70	1116.33	4.633 (28.769)	-2.274 (28.894)	406	730.32	857.24	124.258*** (29.916)	123.823*** (30.838)
Average number of families in catchment area	415	172.45	185.66	13.555** (5.458)	12.706** (5.66)	406	126	153.69	26.827*** (5.223)	26.54*** (5.39)

For peer review only

For peer review only - http://bmjopen.bmj.com/site/about/guidelines.xhtml

Indicators	Bihar					Madhya Pradesh				
	N	Mean (Comparison)	Mean (Intervention)	Mean Diff. (Std. Err.)		N	Mean (Comparison)	Mean (Intervention)	Mean Diff. (Std. Err.)	
				District Pairs	Village Pairs				District Pairs	Village Pairs
Average number of children aged 0-5 months in catchment area	403	10.56	11.10	0.551 (1.12)	0.606 (1.194)	404	5.64	9.32	3.697*** (1.232)	3.655*** (1.269)
Average number of children aged 6-12 months in catchment area	393	17.64	17.26	-0.307 (1.559)	-0.283 (1.757)	400	7.62	9.58	1.973*** (0.698)	1.984*** (0.75)
Average number of children aged 12-24 months in catchment area	387	27.78	27.80	0.208 (2.256)	0.017 (2.446)	398	15	19.98	5.011*** (1.085)	5.34*** (1.164)
Average number of pregnant women in catchment area	417	12.30	12.36	0.053 (0.458)	0.068 (0.464)	406	7.39	7.71	0.306 (0.576)	0.237 (0.604)
Average number of lactating women in catchment area	417	12.05	11.10	-0.948* (0.513)	-0.976* (0.508)	406	5.86	7.36	1.503*** (0.405)	1.444*** (0.43)
**Anganwadi Center (AWC) Characteristics**										
% AWCs with pucca construction	420	0.55	0.52	-0.033 (0.047)	-0.033 (0.047)	412	0.8	0.85	0.04 (0.037)	0.039 (0.039)
% AWCs with an Anganwadi Helper	420	0.91	0.91	-0.004 (0.028)	-0.005 (0.028)	412	0.96	0.97	0.005 (0.019)	0.005 (0.019)
% AWCs with drinking water on the premises	420	0.43	0.46	0.029 (0.049)	0.029 (0.049)	412	0.68	0.58	-0.098** (0.046)	-0.108** (0.048)
% AWCs with a functional toilet on the premises	420	0.11	0.18	0.07** (0.035)	0.062* (0.034)	412	0.39	0.29	-0.1** (0.045)	-0.103** (0.045)
% AWCs with electricity	420	0.16	0.17	0.003 (0.036)	0.005 (0.037)	412	0.21	0.15	-0.055 (0.038)	-0.054 (0.038)
% AWCs with Salter scales to weigh children	420	0.59	0.74	0.151*** (0.044)	0.148*** (0.044)	412	0.96	0.91	-0.048** (0.024)	-0.049* (0.025)
% AWCs with the new WHO Growth Monitoring charts	420	0.28	0.30	0.026 (0.044)	0.024 (0.048)	412	0.69	0.58	-0.108** (0.046)	-0.113** (0.048)
**Anganwadi Worker (AWW) Characteristics**										

For peer review only

Indicators	Bihar					Madhya Pradesh				
	N	Mean (Comp-arison)	Mean (Interv-ention)	Mean Diff. (Std. Err.)		N	Mean (Comp-arison)	Mean (Interv-ention)	Mean Diff. (Std. Err.)	
				District Pairs	Village Pairs				District Pairs	Village Pairs
Average age in completed years	420	39.11	37.54	-1.565** (0.744)	-1.555** (0.756)	412	39.45	40.57	1.124 (0.868)	1.069 (0.888)
Average education level in years	420	11.53	11.81	0.283 (0.201)	0.278 (0.203)	412	10.12	10.86	0.739** (0.334)	0.75** (0.336)
% AWWs who belong to Scheduled Castes or Scheduled Tribe categories	420	0.17	0.21	0.041 (0.038)	0.043 (0.039)	412	0.53	0.43	-0.102*** (0.039)	-0.108** (0.042)
% AWWs who own a bank account	420	0.99	1.00	0.01 (0.009)	0.01 (0.01)	412	1	1	0.005 (0.005)	0.005 (0.005)
% AWWs who are currently married	420	0.93	0.96	0.024 (0.022)	0.024 (0.023)	412	0.82	0.87	0.05 (0.036)	0.049 (0.038)
% AWWs who are presently engaged in any other work to earn additional income	420	0.01	0.06	0.042** (0.018)	0.043** (0.017)	412	0.03	0.12	0.087*** (0.026)	0.088*** (0.026)
% AWWs who received job training on joining	420	0.99	0.97	-0.014 (0.014)	-0.014 (0.014)	382	0.95	0.93	-0.015 (0.025)	-0.017 (0.028)
% AWWs who report ownership of a smart phone	420	0.03	0.93	0.895*** (0.021)	0.895*** (0.021)	412	0.23	0.94	0.713*** (0.034)	0.711*** (0.036)
**% AWWs who report providing the following services in the last calendar month**										
Take Home Ration to pregnant women, lactating women, and children < 3 years	420	0.70	0.19	-0.509*** (0.041)	-0.507*** (0.041)	412	0.98	0.96	-0.014 (0.017)	-0.015 (0.018)
Home Visits	420	0.86	0.89	0.034 (0.031)	0.033 (0.031)	412	0.98	0.98	0 (0.014)	0 (0.014)
Iron supplements to pregnant women	420	0.13	0.30	0.174*** (0.039)	0.172*** (0.04)	412	0.87	0.87	0.006 (0.033)	0 (0.033)
Iron supplements to children aged 6 months-2 years	420	0.91	0.96	0.048** (0.024)	0.048** (0.023)	412	0.98	0.93	-0.048** (0.02)	-0.049** (0.019)

For peer review only

Indicators	Bihar					Madhya Pradesh				
	N	Mean (Comp-arison)	Mean (Interv-ention)	Mean Diff. (Std. Err.)		N	Mean (Comp-arison)	Mean (Interv-ention)	Mean Diff. (Std. Err.)	
				District Pairs	Village Pairs				District Pairs	Village Pairs
Vaccination	420	0.16	0.19	0.031 (0.036)	0.029 (0.037)	412	0.6	0.7	0.096** (0.047)	0.098** (0.047)
Vitamin A supplements	420	0.06	0.06	-0.005 (0.023)	-0.005 (0.024)	412	0.71	0.69	-0.021 (0.044)	-0.02 (0.047)
Growth monitoring	420	0.44	0.52	0.076 (0.047)	0.072 (0.045)	412	0.96	0.95	-0.009 (0.021)	-0.01 (0.022)
Pre-school education to children aged 3 years and above	420	0.99	1.00	0.014* (0.008)	0.014* (0.008)	412	0.99	0.98	-0.01 (0.012)	-0.01 (0.012)
**AWW Knowledge**										
% AWWs who knew at least half the correct messages and checks to be given to women in last trimester of pregnancy	420	0.26	0.26	-0.008 (0.041)	-0.005 (0.041)	412	0.29	0.25	-0.041 (0.043)	-0.044 (0.043)
% AWWs who knew at least half the correct messages and checks to be given to women who have recently delivered a child	420	0.09	0.09	0.004 (0.026)	0.005 (0.028)	412	0.12	0.06	-0.059** (0.026)	-0.059** (0.026)
% AWWs who knew at least half the correct messages and checks for women with a child aged 6 months	420	0.13	0.16	0.031 (0.032)	0.033 (0.034)	412	0.23	0.16	-0.069* (0.037)	-0.069* (0.039)
% AWWs with knowledge* on Pregnancy care and Birth Preparedness, as per the technical quiz	420	0.77	0.66	-0.107** (0.042)	-0.11*** (0.04)	412	0.64	0.65	0.004 (0.046)	0.01 (0.045)
% AWWs with knowledge on New Born Care in the technical quiz	420	0.33	0.26	-0.065 (0.043)	-0.067 (0.043)	412	0.34	0.38	0.045 (0.047)	0.049 (0.046)

For peer review only

Indicators	Bihar					Madhya Pradesh				
	N	Mean (Comp-arison)	Mean (Interv-ention)	Mean Diff. (Std. Err.)		N	Mean (Comp-arison)	Mean (Interv-ention)	Mean Diff. (Std. Err.)	
				District Pairs	Village Pairs				District Pairs	Village Pairs
% AWWs with knowledge on Family Planning in the technical quiz	420	0.84	0.88	0.039 (0.032)	0.038 (0.034)	412	0.86	0.9	0.04 (0.031)	0.039 (0.033)
% AWWs with knowledge on Breastfeeding in the technical quiz	420	0.85	0.85	0.001 (0.034)	0 (0.033)	412	0.84	0.87	0.031 (0.034)	0.029 (0.035)
% AWWs with knowledge on Complementary feeding in the technical quiz	420	0.99	0.99	0 (0.01)	0 (0.01)	412	0.99	0.97	-0.019 (0.015)	-0.02 (0.016)
% AWWs who correctly answered at least half the questions in the Growth Monitoring quiz	420	0.45	0.45	-0.001 (0.042)	-0.005 (0.042)	412	0.68	0.72	0.042 (0.044)	0.044 (0.044)

For peer review only

Balance in Primary and Secondary Outcome Indicators

Indicators	Bihar					Madhya Pradesh				
	N	Mean (Comparison)	Mean (Intervention)	Mean Diff. (Std. Err.)		N	Mean (Comparison)	Mean (Intervention)	Mean Diff. (Std. Err.)	
				District Pairs	Village pairs				District Pairs	Village pairs
**Primary Outcomes**										
% of beneficiaries with 0-6m child who report at least two home visits by Anganwadi Workers (AWW) during their last trimester of pregnancy	810	0.05	0.08	0.026 (0.018)	0.031** (0.015)	757	0.2	0.23	0.036 (0.032)	0.035 (0.028)
% of beneficiaries with 0-6m child who received at least half the messages as per Integrated Child Development Scheme (ICDS) guidelines during their last trimester of pregnancy	810	0.08	0.12	0.038* (0.022)	0.039** (0.019)	757	0.2	0.23	0.037 (0.031)	0.027 (0.027)
% beneficiaries with 0-6m child who received any visit from AWW within 24 hours after birth	810	0.06	0.05	-0.016 (0.017)	-0.018 (0.015)	757	0.07	0.1	0.032 (0.021)	0.04** (0.018)
% beneficiaries with 0-6m child who received at least 2 visits within the first week of birth	810	0.03	0.03	0.002 (0.012)	0.001 (0.01)	757	0.04	0.05	0.003 (0.016)	0.004 (0.014)
% beneficiaries with 0-6m child who received appropriate number of visits as per ICDS guidelines within first six months of birth	810	0.02	0.03	0.014 (0.012)	0.013 (0.011)	757	0.04	0.04	0 (0.016)	0.002 (0.013)
% beneficiaries with 6-12m child who received appropriate number of visits as per ICDS guidelines since their child turned 6 months	444	0.03	0.05	0.023 (0.018)	0.025 (0.018)	465	0.14	0.12	-0.027 (0.031)	-0.03 (0.033)

For peer review only

Indicators	Bihar					Madhya Pradesh				
	N	Mean (Comp-arison)	Mean (Interv-ention)	Mean Diff. (Std. Err.)		N	Mean (Comp-arison)	Mean (Interv-ention)	Mean Diff. (Std. Err.)	
				District Pairs	Village pairs				District Pairs	Village pairs
% of beneficiaries with 6-12m child who received at least half the messages as per ICDS guidelines since their child turned 6 months	444	0	0.01	0.005 (0.008)	0.005 (0.008)	465	0.07	0.05	-0.019 (0.022)	-0.019 (0.022)
% beneficiaries with 12-24m child who received at least 2 visits since their child turned 12 months	431	0.97	0.96	-0.009 (0.018)	-0.011 (0.017)	431	0.94	0.91	-0.035 (0.025)	-0.03 (0.028)
% beneficiaries with child 0-24m who reported that their child was weighed by AWW in the last 3 months	1685	0.16	0.24	0.085*** (0.024)	0.085*** (0.021)	1653	0.54	0.6	0.057** (0.028)	0.057*** (0.022)
% beneficiaries with child 0-24m who reported that AWW discussed their child's weight and nutritional status in the last 3 months	1685	0.11	0.17	0.063*** (0.02)	0.063*** (0.017)	1653	0.37	0.39	0.021 (0.029)	0.021 (0.021)
**Secondary Outcomes**										
**Knowledge about Pregnancy care, Birth preparedness, Health and Nutrition**										
% beneficiaries with 0-6m child who are aware of at least half of the things a pregnant woman should do to take care of herself and baby during pregnancy	810	0.57	0.52	-0.052 (0.04)	-0.047 (0.034)	757	0.59	0.53	-0.06 (0.037)	-0.06** (0.03)
% beneficiaries with 0-6m child who are aware of at least half of the infant danger signs	810	0.04	0.03	-0.016 (0.014)	-0.013 (0.012)	757	0.02	0.06	0.04*** (0.015)	0.041*** (0.013)

For peer review only

Indicators	Bihar					Madhya Pradesh				
	N	Mean (Comp-arison)	Mean (Interv-ention)	Mean Diff. (Std. Err.)		N	Mean (Comp-arison)	Mean (Interv-ention)	Mean Diff. (Std. Err.)	
				District Pairs	Village pairs				District Pairs	Village pairs
% of beneficiaries with 0-6m child who know how soon an infant should be breastfed after birth of the child	810	0.7	0.72	0.022 (0.033)	0.025 (0.027)	757	0.67	0.72	0.055 (0.034)	0.055* (0.029)
% beneficiaries with 0-6m child who know that infants should not be given water when the infant is less than 6m old	810	0.43	0.35	-0.086** (0.036)	-0.091*** (0.03)	757	0.35	0.37	0.023 (0.036)	0.032 (0.031)
**Services received during pregnancy**										
% currently pregnant women who registered their pregnancy with either Accredited Social Health Activist (ASHA), Auxiliary Nurse Midwife (ANM), AWW, or Govt. Center	1458	0.95	0.97	0.017 (0.011)	0.021** (0.009)	983	0.97	0.99	0.01 (0.009)	0.008 (0.007)
% currently pregnant women who received ANY Antenatal Care (ANC) check-up during their pregnancy	1458	0.83	0.85	0.024 (0.023)	0.029 (0.02)	983	0.93	0.98	0.051*** (0.015)	0.038*** (0.011)
% currently pregnant women who report attending at least one Villahe Health and Nutrition Day (VHND) during their pregnancy	1458	0.47	0.5	0.029 (0.035)	0.022 (0.028)	983	0.65	0.57	-0.089** (0.036)	-0.086*** (0.033)
**Ante-natal Care (ANC)**										
% beneficiaries with 0-24m child who received at least 4 ANC check-ups during pregnancy	1685	0.25	0.2	-0.05** (0.023)	-0.05*** (0.017)	1653	0.52	0.51	-0.003 (0.028)	-0.005 (0.022)
% beneficiaries with 0-24m child who consumed Iron Folic Acid for more than 90 days during pregnancy	1685	0.12	0.12	0.001 (0.017)	0.001 (0.013)	1653	0.54	0.49	-0.054** (0.027)	-0.055** (0.022)

For peer review only

Indicators	Bihar					Madhya Pradesh				
	N	Mean (Comp-arison)	Mean (Interv-ention)	Mean Diff. (Std. Err.)		N	Mean (Comp-arison)	Mean (Interv-ention)	Mean Diff. (Std. Err.)	
				District Pairs	Village pairs				District Pairs	Village pairs
% beneficiaries with 0-24m child who received 2 Tetanus Toxoid (TT) injections during pregnancy	1685	0.84	0.81	-0.033 (0.02)	-0.032** (0.016)	1653	0.81	0.87	0.061*** (0.02)	0.061*** (0.016)
% beneficiaries with 0-24m child who delivered at a health facility	1685	0.73	0.65	-0.079*** (0.028)	-0.079*** (0.021)	1653	0.81	0.81	0.008 (0.022)	0.007 (0.016)
**Beneficiary practices post-delivery**										
% beneficiaries with 0-24m child who breastfed their child within an hour of birth	1685	0.76	0.72	-0.04 (0.025)	-0.04** (0.018)	1653	0.72	0.75	0.024 (0.026)	0.022 (0.019)
% beneficiaries with 0-24m child who had applied nothing to the cord after birth	1685	0.57	0.59	0.025 (0.028)	0.026 (0.021)	1653	0.6	0.6	0.006 (0.027)	0.007 (0.021)
**AWW Home Visits**										
% currently pregnant women who report ANY home visits by AWW during pregnancy	1458	0.23	0.3	0.065** (0.028)	0.057** (0.023)	983	0.39	0.42	0.025 (0.039)	0.012 (0.034)
% currently pregnant women who report that the AWW discussed or checked their Iron Folic Acid intake during a home visit	1458	0.1	0.19	0.091*** (0.024)	0.085*** (0.019)	983	0.36	0.39	0.034 (0.038)	0.024 (0.033)
% currently pregnant women who report that the AWW asked about ANC check-ups, immunization, blood tests, etc. during a home visit	1458	0.13	0.19	0.061** (0.026)	0.047** (0.021)	983	0.31	0.37	0.059* (0.035)	0.053* (0.031)

For peer review only

Indicators	Bihar					Madhya Pradesh				
	N	Mean (Comparison)	Mean (Intervention)	Mean Diff. (Std. Err.)		N	Mean (Comparison)	Mean (Intervention)	Mean Diff. (Std. Err.)	
				District Pairs	Village pairs				District Pairs	Village pairs
% beneficiaries with 0-6m child who report that AWW discussed child's birth weight and health status during home visit	810	0.04	0.06	0.021 (0.016)	0.021 (0.014)	757	0.12	0.13	0.013 (0.025)	0.012 (0.021)
% beneficiaries with 6-24m child who reported AWWs discussing child's weight and adequacy of growth during home visit	875	0.03	0.07	0.042*** (0.016)	0.042*** (0.014)	896	0.21	0.14	-0.064** (0.028)	-0.063*** (0.023)
**Family Planning**										
% beneficiaries with 0-24m child who received advice on family planning needs, methods, resources	1685	0.02	0.04	0.023*** (0.008)	0.023*** (0.007)	1653	0.08	0.09	0.004 (0.015)	0.004 (0.011)
% beneficiaries with 0-24m child who is currently using family planning	1685	0.3	0.27	-0.023 (0.025)	-0.023 (0.019)	1653	0.37	0.34	-0.023 (0.025)	-0.023 (0.019)
**Take Home Ration (THR)**										
% currently pregnant beneficiaries who received THR from the AWC during pregnancy	1458	0.53	0.4	-0.131*** (0.034)	-0.127*** (0.027)	983	0.9	0.88	-0.026 (0.022)	-0.023 (0.019)
% beneficiaries with 6-24m child who received THR in the first 6 months after birth of child	875	0.71	0.58	-0.129*** (0.035)	-0.123*** (0.03)	896	0.91	0.92	0.007 (0.019)	0.008 (0.015)
% beneficiaries with 6-24m child who received THR for child > 6m	875	0.5	0.43	-0.068* (0.036)	-0.06** (0.03)	896	0.75	0.77	0.026 (0.031)	0.029 (0.025)
% beneficiaries with 0-24m child who consumed entire THR themselves during pregnancy	1685	0.23	0.19	-0.038 (0.023)	-0.038** (0.018)	1653	0.33	0.39	0.063** (0.026)	0.062*** (0.02)
**Infant and Young Child Feeding (IYCF) - Knowledge and Practices**										

For peer review only

Indicators	Bihar					Madhya Pradesh				
	N	Mean (Comp-arison)	Mean (Interv-ention)	Mean Diff. (Std. Err.)		N	Mean (Comp-arison)	Mean (Interv-ention)	Mean Diff. (Std. Err.)	
				District Pairs	Village pairs				District Pairs	Village pairs
% beneficiaries with 6-12m child who know the right time to start complementary feeding	444	0.95	0.93	-0.021 (0.023)	-0.02 (0.022)	465	0.88	0.9	0.015 (0.029)	0.01 (0.027)
% infants 0-5m of age who received only breastmilk during the previous day	810	0.6	0.51	-0.087*** (0.033)	-0.088*** (0.028)	757	0.46	0.5	0.032 (0.034)	0.038 (0.03)
% infants 6-8m of age who received solid, semi-solid or soft foods during the previous day	266	0.53	0.55	0.016 (0.06)	0.034 (0.088)	264	0.56	0.62	0.051 (0.06)	0.137 (0.094)
% children 6-24m of age who received foods from >= 4 food groups during the previous day	875	0.12	0.15	0.038* (0.023)	0.04** (0.019)	896	0.17	0.22	0.052* (0.027)	0.054** (0.022)
**Growth Monitoring**										
% beneficiaries with 0-6m child who were aware that growth monitoring for children was one of the services at AWCs	810	0.1	0.14	0.039 (0.026)	0.037* (0.021)	757	0.3	0.21	-0.093*** (0.033)	-0.099*** (0.027)
% beneficiaries with 0-24m child who reported that child birth weight as available	1685	0.67	0.58	-0.08*** (0.028)	-0.081*** (0.021)	1653	0.79	0.81	0.02 (0.021)	0.02 (0.015)
% beneficiaries with 0-24m child whose child's birth weight was verified from Mother-Child Protection (MCP)/Health card	1685	0.16	0.15	-0.011 (0.019)	-0.011 (0.014)	1653	0.24	0.29	0.045* (0.024)	0.043** (0.019)
**Diet and Food security**										

For peer review only

Indicators	Bihar					Madhya Pradesh				
	N	Mean (Comp-arison)	Mean (Interv-ention)	Mean Diff. (Std. Err.)		N	Mean (Comp-arison)	Mean (Interv-ention)	Mean Diff. (Std. Err.)	
				District Pairs	Village pairs				District Pairs	Village pairs
% mothers of 0-24m children who have consumed at least five out of ten defined food groups the previous day or night	1685	0.16	0.18	0.015 (0.021)	0.015 (0.016)	1653	0.14	0.16	0.015 (0.021)	0.016 (0.016)
% households which are food secure[1]	1685	0.79	0.7	-0.086*** (0.023)	-0.086*** (0.018)	1653	0.76	0.75	-0.01 (0.023)	-0.011 (0.018)
**Immunization**										
% beneficiaries whose child aged 0-24m ever received any immunizations	1685	0.97	0.97	0 (0.009)	0 (0.007)	1653	0.97	0.98	0.01 (0.008)	0.009 (0.006)
% beneficiaries with 0-24m child who are aware that AWW records details of immunization	1685	0.26	0.3	0.039 (0.025)	0.039** (0.018)	1653	0.32	0.31	-0.016 (0.027)	-0.016 (0.021)
% beneficiaries with 0-24m child who reported that they had MCP cards	1685	0.86	0.86	-0.005 (0.018)	-0.005 (0.014)	1653	0.74	0.91	0.174*** (0.022)	0.175*** (0.017)
% beneficiaries with 0-24m child whose MCP cards were available at home or at Anganwadi Center, and were at least partially filled	1685	0.63	0.65	0.021 (0.025)	0.021 (0.02)	1653	0.54	0.69	0.15*** (0.027)	0.149*** (0.021)
% beneficiaries whose child aged 12-24m is fully immunized	431	0.31	0.35	0.041 (0.045)	0.043 (0.044)	431	0.31	0.4	0.096** (0.044)	0.097** (0.046)
% beneficiaries whose child aged 12-24m is fully immunized (among the subset of beneficiaries with available MCP cards)	293	0.43	0.52	0.083 (0.059)	0.059 (0.082)	268	0.53	0.57	0.069 (0.062)	0.132 (0.096)

[1] Food and Agricultural Organization (FAO) "Food Insecurity Experience Scale Survey Module (FIES-SM) – Household Referenced" (http://www.fao.org/3/a-bl404e.pdf)

For peer review only

Effects of an mHealth intervention for community health workers on maternal and child nutrition and health service delivery in India: Study protocol for a quasi-experimental mixed methods evaluation

Journal:	*BMJ Open*
Manuscript ID	bmjopen-2018-025774.R1
Article Type:	Protocol
Date Submitted by the Author:	12-Dec-2018
Complete List of Authors:	Nimmagadda, Sneha; NEERMAN, Center for Causal Research and Impact Evaluation<br>Gopalakrishnan, Lakshmi; NEERMAN, Center for Causal Research and Impact Evaluation<br>Avula, Rasmi; International Food Policy Research Institute<br>Dhar, Diva; Bill and Melinda Gates Foundation India<br>Diamond-Smith, Nadia; University of California San Fransisco<br>Fernald, Lia; University of California, Berkeley, School of Public Health<br>Jain, Anoop; University of California, Berkeley, School of Public Health<br>Mani, Sneha; International Food Policy Research Institute<br>Menon, Purnima; International Food Policy Research Institute<br>Nguyen, Phuong Hong; International Food Policy Research Institute<br>Park, Hannah; University of California San Fransisco<br>Patil, Sumeet; NEERMAN, Center for Causal Research and Impact Evaluation<br>Singh, Prakarsh; Institute of Labour Economics (IZA)<br>Walker, Dilys; University of California San Fransisco
**Primary Subject Heading**:	Global health
Secondary Subject Heading:	Nutrition and metabolism, Public health, Research methods, Health policy
Keywords:	mHealth, child health and nutrition, impact evaluation, Quality in health care < HEALTH SERVICES ADMINISTRATION & MANAGEMENT

SCHOLARONE™
Manuscripts

Effects of an mHealth intervention for community health workers on maternal and child nutrition and health service delivery in India: Study protocol for a quasi-experimental mixed methods evaluation

For peer review only

Authors

Authors	Affiliation	Contact Information
Nimmagadda, Sneha	Network for Engineering, Economics, Research and Management (NEERMAN)	sneha.n@neerman.org
Gopalakrishnan, Lakshmi	Network for Engineering, Economics, Research and Management (NEERMAN)	lakshmi.g@neerman.org
Avula, Rasmi, PhD	International Food Policy Research Institute (IFPRI)	r.avula@cgiar.org
Dhar, Diva	Bill and Melinda Gates Foundation	Diva.Dhar@gatesfoundation.org
Diamond-Smith, Nadia, PhD	University of California at San Francisco (UCSF)	Nadia.Diamond-Smith@ucsf.edu
Fernald, Lia, PhD	University of California at Berkeley (UCB)	fernald@berkeley.edu
Jain, Anoop	University of California at Berkeley (UCB)	anoopjain@berkeley.edu
Mani, Sneha	International Food Policy Research Institute (IFPRI)	s.mani@cgiar.org
Menon, Purnima, PhD	International Food Policy Research Institute (IFPRI)	p.menon@cgiar.org
Nguyen, Phuong, PhD	International Food Policy Research Institute (IFPRI)	P.H.Nguyen@cgiar.org
Park, Hannah	University of California at San Francisco (UCSF)	hannah.park@ucsf.edu
Patil, Sumeet, PhD	Network for Engineering, Economics, Research and Management (NEERMAN)	srpatil@neerman.org
Singh, Prakarsh, PhD	Institute of Labour Economics (IZA)	prakarsh9@gmail.com
Walker, Dilys, PhD	University of California at San Francisco (UCSF) and University of California at Berkeley (UCB)	dilys.walker@ucsf.edu

Corresponding author: Patil, Sumeet

For peer review only - http://bmjopen.bmj.com/site/about/guidelines.xhtml

ABSTRACT

Introduction: Millions of children in India still suffer from poor health and under-nutrition, despite substantial improvement over decades of public health programs. The Anganwadi centers under the Integrated Child Development Scheme (ICDS) provide a range of health and nutrition services to pregnant women, children <6y and their mothers. However, major gaps exist in the ICDS service delivery. The government is currently strengthening the ICDS through an mHealth intervention called Common Application Software (ICDS-CAS), installed on smart phones, with accompanying multi-level data dashboards. This system is intended to be a job aid for frontline workers, supervisors and managers, and aims to ensure better service delivery and supervision, and enable real time monitoring and data-based decision making. However, there is little to no evidence on the effectiveness of such large-scale mHealth interventions integrated with public health programs in resource constrained settings on the service delivery and subsequent health and nutrition outcomes.

Methods and analysis: This study uses a village-matched controlled design with repeated cross-sectional surveys to evaluate whether ICDS-CAS can enable more timely and appropriate services to pregnant women, children <12 months and their mothers, compared to the standard ICDS programme. The study will recruit approximately 1500 Anganwadi workers and 6000+ mother-child dyads from 400+ matched pairs of villages in Bihar and Madhya Pradesh. The primary outcomes are the proportion of beneficiaries receiving (a) adequate number of home visits and (b) appropriate level of counselling by the Anganwadi workers. Secondary outcomes are related to improvements in other ICDS services, and knowledge and practices of the Anganwadi workers and beneficiaries.

Ethics: Ethical oversight is provided by the Committee for the Protection of Human Subjects at the University of California at Berkeley, and the Suraksha Independent Ethics Committee in India. The results will be published in peer-reviewed journals and analysis data will be made public.

Trial registration number: https://doi.org/10.1186/ISRCTN83902145

For peer review only

STRENGTHS AND LIMITATIONS

- The study can provide important evidence on whether and the extent to which a large-scale *mHealth* intervention can improve the maternal and child health and nutrition service delivery by the world's largest child development program in India.
- Application of the gold-standard cluster-randomized controlled trial design was not possible because of pre-determined program assignment and the rapid roll-out of the program. Therefore, to find attributable impacts, this evaluation settles for a scientifically less robust but practicable quasi-experimental design consisting of matched control villages and repeated cross-sectional measurements.
- Measurement biases may exist because blinding is not possible and primary outcomes are measured subjectively via interview-based recall or observations.
- Higher order impacts may be underestimated as the follow-up period of <12m may be too short for the intervention to stabilize.

For peer review only

INTRODUCTION

Background and Problem Context

Millions of children in India continue to suffer from poor health and under-nutrition, despite decades of government programs aiming to reduce this burden and some impressive gains through these years. In 2015-16, 36% of children under 5 years of age were underweight, 38% were stunted, and 21% were wasted as per the National Family Health Survey (NFHS-4), and these numbers represent only modest improvements over the past decade. Micronutrient deficiencies are widespread, with more than 58% of preschool children suffering from iron deficiency anemia. Infant and neonatal mortality rates also remain high at 41 and 30 per 1000 live births respectively, despite substantive reductions over past decades.[1]

The Integrated Child Development Services (ICDS),launched in 1975, is one of India's national flagship programs to support the health, nutrition, and development needs of children below 6 years of age, and pregnant and lactating women, through a network of Anganwadi Centers (AWCs), each typically serving a population of 800-1000.[2,3] Early observational studies found that the ICDS is associated with better coverage and delivery of services related to nutrition, health care, and pre-school education, and improved maternal and child nutrition.[4–6] Using the NFHS data from 2005-06, Kandpal[7] and Jain[8] found that ICDS is associated with small to modest improvements in child health and nutrition especially among the most vulnerable populations.

However, several reviews and evaluations of the ICDS over the past 18 years have also found persistent gaps including inadequate infrastructure at the AWC, Anganwadi worker (AWW) service delivery issues (e.g., poor quality supplementary food, few home visits, no counselling etc.), human resource issues (e.g., vacancies, increasing range of duties expected of the AWWs, inadequate training of AWWs, limited supervision, etc.), and poor data management (e.g., irregularities in record keeping at AWCs, ineffective monitoring of service delivery, etc.).[3,4,9–11] The most recent NFHS (2015-16) also highlights the gaps in the ICDS service delivery. Only about 59% of the children under 6 years received any service from an AWC, 53% received supplementary food services, and 47% were weighed. Similarly, only 60% of mothers received any AWC services during pregnancy, and only 54% received any service during the breastfeeding phase.[1]

With a goal to improve the functioning of the ICDS, the Government of India launched the ICDS Systems Strengthening and Nutrition Improvement Programme (ISSNIP) in 2012 which focused on infrastructure upgradation and training of AWWs to build their knowledge on health and nutrition topics under the Incremental Learning Approach. At the same time, a pilot-scale mHealth intervention to improve ICDS service delivery was implemented in Bihar between 2012 and 2013. A randomized controlled trial of this intervention found a significant increase in the proportion of beneficiaries receiving visits from frontline workers at different life stages - last trimester of pregnancy (42% vs. 52%), first week after delivery (60% vs. 73%), and complementary feeding stage >5 months after delivery (36% vs. 45%).[12] The intervention also significantly increased the proportion of beneficiaries receiving at least three ANC visits

For peer review only

(29% vs. 50%), the proportion of beneficiaries consuming at least 90 Iron Folic Acid tablets during pregnancy (11% vs. 17%), the proportion of mothers immediately breastfeeding after birth (62% vs. 76%) and the proportion of mothers starting complementary feeding at the right time (32% vs. 41%). Subsequently, the ISSNIP was restructured in 2015 by integrating ICDS in seven states with an at-scale *mHealth* intervention called Common Application Software (ICDS-CAS), installed on smart phones, and with accompanying multi-level data dashboards. This system is intended to be a job aid for frontline workers, supervisors and managers, and aims to ensure better service delivery and supervision, and enable real time monitoring and data-based decision making.

While there is a growing body of evidence on the effectiveness of mHealth interventions, it almost entirely consists of small-scale studies or pilot interventions under well controlled settings, and often of poor research quality. For example, a systematic review examining 17 studies set in low- and middle-income countries found that small scale mHealth interventions, particularly those delivered using SMS, were associated with increased utilization of health care, including uptake of recommended prenatal and postnatal care consultation, skilled birth attendance, and vaccination, but only two of these studies were graded as being at *low risk of bias*.[13] Barnett and Gallegos[14] reviewed nine studies that assessed the impact of using of mobile phones for health and nutrition surveillance, and found that while the available evidence suggests that mobile phones may play an important role in nutrition surveillance by reducing the time required to collect data and by enhancing data quality, the available evidence is of poor methodological quality, generally based on small pilot studies and mainly focuses on feasibility issues. Another recent systematic review of 25 studies found evidence that mobile tools helped community health workers improve the quality of care provided, efficiency of services, and capacity for program monitoring.[15] However, most of these studies were pilots and provided little or no information about the effectiveness of mHealth interventions when integrated with large-scale public health programs.

This study seeks to address this critical gap in the evidence base in the context of the largest public health and nutrition program in the world, the ICDS, with 1.4 million AWCs serving at the grassroots levels across India. The impact evaluation is conducted in two large states in India, Madhya Pradesh and Bihar, and uses a quasi-experimental, matched-controlled pre-post measurement design. The overall evaluation framework consists of additional components such a process evaluation, a technology evaluation and an economic evaluation.

This evaluation is also timely as India launched the National Nutrition Mission on 8th March 2018 with the goal of reducing malnutrition in a phased manner across entire India and subsumed ISSNIP and ICDS-CAS under it.[16] Therefore, ISSNIP and ICDS-CAS are poised to be scaled up rapidly to reach almost the entire population of India through1.4 million AWCs by 2020. This scale-up effort will be informed by robust evidence on the effectiveness of the mHealth intervention, as well as on how its implementation can be improved.

The ICDS-CAS Intervention

For peer review only

Currently, the ICDS-CAS intervention is being implemented at scale in seven states covering over 107,000 AWCs, and through them, a population of 9.8 million registered beneficiaries. The intervention consists of two components as follows.[17]

(1) An android CAS application and smart phones for AWWs and Lady Supervisor: The CAS app was developed on an open source mobile platform (CommCare). The app digitizes and automates ten of the eleven ICDS paper registers maintained by AWWs, enables name-based tracking of beneficiaries, prioritizes home visits at critical life stages through a home visit-scheduler, improves record keeping and retrieval of growth or nutrition status of children, helps track immunization, monitors timeliness and quality of different services delivered by AWWs, and includes checklists and videos as job aids. A lady supervisor typically manages a cluster of 10-20 AWCs and the CAS App is expected to help her monitor AWWs remotely, assess quality of service delivery, and serves as a job aid to train AWWs. The app is installed on new smartphones that are provided to the AWWs and supervisors. Both AWWs and supervisors are trained on the use of Apps and how the features help them improve service delivery. Helpdesks at block and district levels for technical support are also established.

The CAS app is especially expected to improve home visit service delivery by the AWW through improved channels of information (easy access to past records of the beneficiary for customized messaging, educational animation videos as a job aid, life-stage-appropriate checklists for counseling messages) and timely nudges (automatic creation of visit due lists, alerts for approaching or missed visits, timely intimation of delays to the lady supervisor). Thus, improved home visits in terms of timeliness, frequency, and perhaps, a more effective message delivery mechanism are expected to result in increased knowledge and better recall of correct health and nutrition practices by the beneficiaries and higher demand for related government services. However, for the actual behaviors to change and sustain, supply side constraints must be addressed to meet the demand for services (e.g., adequate supply of supplementary food, adequate provisions of IFA tablets, regular immunization camps, etc.). Such improvements can be expected only in the mid- to long-term because they are beyond the sphere of influence of ICDS-CAS and need more ICDS-wide improvements.

(2) A web-enabled dashboard for real-time monitoring by ICDS officials: Data generated at the AWC-level are aggregated and analyzed via web-enabled dashboards for Child Development Project Officers at the project-level (typically an administrative block with 80-100 AWCs), District Programme Officers, a state's ICDS Directorate, and the Ministry of Women and Child Development (MWCD) at the national-level. For example, the monthly progress reports are prepared manually at the AWC-level and then aggregated to the project-level, which requires weeks to be finalized and reviewed, but the CAS App and dashboards will automate and produce these reports in almost real time. The dashboard infographics are expected to help identify bottlenecks at various levels more efficiently, help prioritize local issues, and allow managers to take data-driven actionable decisions.

Figure 1 presents the ICDS-CAS information flow from the AWC through to the MWCD. The logic model in **Figure 2** summarizes how ICDS-CAS is expected to improve service delivery, and ultimately to improve health and nutritional outcomes in mothers and their children. The listed short-term outcomes are those expected to be achieved in the planned evaluation follow-up period of <12 months. The longer-

For peer review only

term outcomes related to improved health and nutrition will be measured (except improvements in the cognitive abilities) and analyzed from a learning perspective, but these remain aspirational in the context of this evaluation study.

Evaluation Framework and Research Objectives

The ICDS-CAS evaluation framework consists of four components – an impact evaluation, a process evaluation, an economic evaluation, and a technology evaluation. This paper describes the protocol for the impact evaluation in detail to guide the final analysis plan. The other three components are summarized in **Supplementary Figure 1** without binding details because their objectives, scope and methods can change as per the evolving learning needs of policy makers. We intend to analyze and publish the impact evaluation and process evaluation as standalone research articles in peer reviewed journals. The analysis and publication plan for the other components is yet to be determined.

Corresponding to the short-term strategic objective of ICDS-CAS to improve quality and quantity of AWW and beneficiary interactions, the main research questions the impact evaluation seeks to answer are:

1. Does ICDS-CAS improve the timeliness or frequency of home visits by the AWW to pregnant women, infants and their mothers?
2. Does ICDS-CAS improve extent or level of counselling from AWWs to pregnant women and mothers of infants?

METHODS AND ANALYSIS

Study Setting

The ICDS-CAS program is being implemented in 57 districts from seven ISSNIP states in India where the burden of under-nutrition is highest. This evaluation is restricted to two states, Madhya Pradesh (MP) and Bihar, which were selected because of the possibility of selecting an ISSNIP district as a control, willingness of the states to support the evaluation, and the suggestions by the MWCD and the funding agency. **Supplementary Table 1** presents key health and nutrition related indicators for rural villages of India, MP, and Bihar. Both states have a high burden of under-five mortality (69 per 1,000 live births in MP, and 60 in Bihar), stunting (43.6% of children aged 0-5 years in MP, and 49.3% in Bihar), and anemia (>55% of children and pregnant women in both states). The antenatal- and delivery-related indicators are, in general, worse in Bihar, whereas MP has relatively poor demographic, water-sanitation, education, and mortality related indicators– 8.3% of mothers in MP and 3% in Bihar had full antenatal care; 79.5% of households in MP and 98.2% in Bihar had an improved drinking-water source; and 50.2% of children aged 12-23 months in MP and 61.9% in Bihar were fully immunised.

Overview of the Identification Strategy

For peer review only

The attributable effects of ICDS-CAS will be identified using a quasi-experimental matched, controlled design with repeated cross-sectional pre- and post-intervention measurements. This identification strategy is grounded in the Neyman–Rubin potential outcomes model where a matched cohort design can yield unbiased estimates of the causal effects under a strong assumption that all confounders are measured and balanced between the intervention and comparison groups.[18–20] We use a 1:1 nearest neighbour propensity score matching (PSM) method to identify pairs of intervention and comparison villages.[21–25] We plan to identify the effects of ICDS-CAS by comparing post-intervention outcome indicators between the matched groups, while controlling for any pre-intervention or baseline differences in the outcomes averaged at the village level and adjusting for the matched paired design.

Sample Design and Power Calculations

The initial sample size was determined as 400 villages in each arm with one AWW and on average three mother-child dyads in each village to measure a relative effect of 15% in a standardized counterfactual outcome [Normal(0,1)], with a significance level of 0.05, power of 80%, and Intra-Cluster Correlation (ICC) of 0.15. The actual baseline survey sample consisted of 852 villages to account for refusals and loss to follow-up.

After the baseline survey was conducted in June-August 2017 and completely separate from our efforts to design the evaluation, the Indian government decided to include ICDS-CAS in the National Nutrition Mission. Consequently, the original evaluation objectives are being revised to estimate the effects of ICDS-CAS separately for MP and Bihar to draw deeper insights into the heterogeneity of impacts. In case of such an eventuality, the evaluation will need to be powered to detect smaller magnitudes of effects on a few process-related secondary outcomes. We plan to increase the sample power by following the same panel of villages but recruiting almost twice as many participants in each village – up to two AWWs and up to eight mother-child dyads. Assuming 200 pairs of villages and 1200 mothers per arm in each state, the sample will have 80% power at a significance level of 0.05 to detect absolute difference of 5-9 %-points from the counterfactual levels between 10%-50% with ICC between 0.15-0.30. With 400 AWWs per arm in each state, the sample will have 80% power to detect effects of 8-12 %-points for AWW level outcomes from the counterfactual levels of 10%-40% with ICC between 0.25-0.45.

Sampling and Recruitment of Study Participants

Figure 3 summarizes the sampling and recruitment of study participants for the baseline survey. First, we sampled intervention districts and selected geographically and administratively matched comparison districts. Then, we randomly sampled intervention villages in two steps of sampling the blocks and then the villages. Finally, we pair-matched intervention villages with villages from the comparison districts and selected the best matched 426 pairs as discussed next.

Sampling of Intervention and Comparison Districts: We randomly sampled three pairs of geographically and administratively matched intervention and comparison districts which shared a boundary and belonged to the same ICDS division to control for division-level confounders related to ICDS administration and management as well as cultural, social, environmental, and economic factors at the

For peer review only

micro-region scale. Within each state, we first sample three invention districts, and the corresponding control districts were selected *de facto* if only one eligible district was available within the division, or randomly, if multiple comparison districts were available. In MP, the sample purposively included one pair of *tribal-only* districts from an equity-focused evaluation perspective. **Figure 4** depicts the states and districts included in the evaluation.

Selecting Matched Pairs of Treatment and Control Villages: From each selected district, we randomly sampled two blocks in MP and three blocks in Bihar. Next, we randomly sampled 345 villages from six intervention blocks in MP and 315 villages from nine ICDS-CAS blocks in Bihar. In both states, the sample frame was restricted to villages with population of 500 or more to increase the possibility that the villages are sufficiently large to be served by dedicated AWCs. Next, we matched the sampled treatment villages with villages from the paired comparison district using a 1:1 nearest neighbour PSM method using variables from Census 2011[1].[20–23]

Table 1 summarizes the matching performance in terms of reduction in the sum of standardized mean bias after matching. The standardized mean bias was reduced by 61-82% after matching across the six district pairs. The Log Reduction (LR) test statistic before matching was statistically significant (*p*-value <0.001), but became insignificant after matching (*p*-value >0.75), suggesting that both groups are statistically similar on average after the matching procedure. We then selected the best matched village pairs– in terms of the difference in the propensity scores of the matched pairs –35 pairs of villages for each intervention block in MP and 24 pairs of villages for each intervention block in Bihar. Overall, we selected 216 matched pairs (432 villages) in Bihar and 210 matched pairs (420 villages) in MP.

Table 1. Comparison of Matching Performance in Reducing Bias

	Madhya Pradesh			Bihar		
	Ujjain-Dewas	Barwani-Alirajpur	Katni-Jabalpur	Samastipur-Darbhanga	Lakhisarai-Muzaffarpur	Sitamarhi-Jamui
Standardized mean bias - before matching	16.6	27.5	40.3	27	35.4	40.8
Standardized mean bias - after matching	6.5	8	9.9	8.3	6.6	9.7
% reduction in mean bias	61%	71%	75%	69%	81%	76%

[1]*Census 2011 variables used for matching: Distance between village and block headquarters (kilometers); Population; % of SC/ST households; % of villages served by public transport; % of villages connected to a major road; % of villages with a public ration shop; % of villages with a post office; % of villages with a bank; Average proportion of households in a village with a bank account; % of villages with an agricultural society; % of villages with a self-help group; Average proportion of households in a village serviced by closed drainage system; Average proportion of households in a village with improved source of drinking water; Average proportion of households in a village with improved sanitation facility; Average proportion of households in a village using electricity as the main source of light; Average proportion of households in a village with a pucca house; Average household asset index for the village*

For peer review only

p-value of LR test - Before matching	0.000	0.000	0.000	0.000	0.000	0.000
p-value of LR test - After matching	0.929	0.929	0.905	0.788	0.997	0.490
Mean difference in propensity score	0.002	0.072	0.131	0.004	0.046	0.181

Recruitment of Study Participants: In the already conducted baseline survey, we randomly sampled one AWC if more than one AWC existed in a selected village. Using the list of beneficiaries available at the selected AWC as a sample frame, we randomly sampled four mother-child dyads after stratification by life-stages: (1) mother of child aged <3 months, (2) mother of child aged 3 to <6 months, (3) mother of child aged 6 to <12 months, and (4) mother of child aged 12 to <24 months. Additionally, we randomly sampled up to five pregnant women or mothers with a child <3 months for more in-depth analysis of ICDS services during pregnancy and child birth. To measure AWC-level outcomes, we conduct a survey of the selected AWWs as well.

The endline will be a repeated cross-sectional sample where pregnant women and mothers with children<12 months will be recruited using the list of beneficiaries available at the AWC at that time. We do not plan to recruit children older than 12 months at the endline because the ICDS-CAS intervention would be active for <12 months and we can realistically measure the changes only among children who were born after the ICDS-CAS implementation started.

All survey participants are/will be recruited for the study after being administered informed consent as per the IRB-approved protocol. Additional assent is taken just before taking anthropometric measurements (height and weight) of the children.

Patient and Public Involvement

This research was done without patient or public involvement in conceptualization, design, implementation, analysis, manuscript development or dissemination.

Primary Outcomes

The primary outcomes to assess the effectiveness of ICDS-CAS compared to the standard ISSNIP and ICDS are:

1. The proportion of pregnant women and mothers of children <12 months who received adequate number of home visits by the AWW in the past three months (adequate number will be the minimum number of visits a respondent must receive as per the ICDS guidelines for the current life-stage/age. Additionally, we will use, as a supporting indicator, the number of visits as a continuous outcome indicator.[26]); and

For peer review only - http://bmjopen.bmj.com/site/about/guidelines.xhtml

2. The proportion of pregnant women and mothers of children <12 months who received appropriate extent or level of counselling from the AWW during their interactions (at home, at AWCs, or in other settings) in the past three months (appropriate level of counselling will be a recall of at least half of the correct messages/counselling that a respondent should receive as per the ICDS guidelines for the current life-stage/age. Additionally, we will use, as a supporting indicator, number of correct messages or services recalled by the respondent as a continuous outcome.[26]).

Secondary Outcomes

Several outputs and outcomes according to the logic model presented in **Figure 2** are secondary outcomes in this evaluation study as discussed before. These include outcomes related to supervisory and capacity building support to AWWs, infrastructure and supplies at the AWC, AWW level outcomes (motivation, satisfaction, knowledge, time allocation for services and record keeping, time allocation for service delivery, number of beneficiaries served), and additional ICDS services that can be improved by ICDS-CAS but also critically dependent on other external factors (growth monitoring of children, provision of IFA and supplemental nutrition, immunization tracking, referrals, etc.). We will also measure higher order but distal or aspirational outcomes related to knowledge, practices, health and nutrition at the beneficiary level.

Outcome Measurements

All beneficiary- and AWW-level outcomes will be measured through structured interviews, with verification of registers and documents wherever possible. The household instruments were developed using standard questions from the WHO and Demographic and Health Survey frameworks, and capture information about the demographic and socio-economic characteristics of the household, birth history and family planning, pregnancy care, awareness and utilization of AWC services at different life-stages, IYCF practices, immunization, knowledge on health and nutrition, and child health and nutritional outcomes (child growth). The AWW instrument captures information about coverage of beneficiaries, service delivery, supervisory support, work incentives and motivation, training received, time allocation, knowledge, and infrastructure and supplies at the AWC. The interviews are administered in *Hindi* on android tablet-based SurveyCTO™ platform.

Analysis Plan

The effects of the ICDS-CAS intervention will be estimated as,

$$Y_{ij,\,t=1} = \beta_0 + \beta_1 \,.\, T_j + Pair\ ID_K + \overline{Y}_{j,t=0} + \varepsilon$$

where,

$Y_{ij,\,t=1}$ = an outcome of interest for beneficiary *i* in village *j* at endline (*t*=1);

T_j = indicator variable which is 1 for intervention and 0 for comparison villages;

$Pair\ ID_K$ = Fixed effects to account for *k* pairs of matched villages;

$\overline{Y}_{j,t=0}$ = average pre-intervention or baseline (t = 0) level of the outcome Y in the village;

ε = the error term of the model; and

β_1 = the effect of the intervention on outcome Y.

We will adjust for the pre-intervention average level of the outcome in a village ($\bar{Y}_{j,t=0}$) to control for, *at least*, the measured or unmeasured *time invariant* village- and higher-level confounders. To the extent the endline questionnaires or sampling are different from those in the baseline, the construction of the outcome indicators in the baseline and endline may differ slightly, but such differences (if any) will not affect the interpretation of impact parameters estimated using the above model specification. Additionally, as a consistency check, we will estimate adjusted treatment effects that control for any observed imbalance in important baseline covariates. We may additionally control for covariates that can help increase the precision of impact estimates. We do not plan to correct the *p*-values for multiple comparisons because we only test a limited number of indicators for the primary outcomes. However, for secondary outcomes when multiple indicators related to a topic or theme are compared, we will adjust the *p*-values for multiple comparisons. We also plan to estimate the effects by adjusting for pairing only at the district level and not at the village-level if accounting for village matching results in substantial sample loss. All analyses will be done in STATA, documented in a DO file, and replicated by two different analysts.

Baseline Balance

Supplementary Table 2 and Supplementary Table 3 present the baseline balance at AWC, household and individual beneficiary levels. The balance is assessed in terms of group mean difference after adjusting for the pairing of villages. As a robustness check, the group mean differences by adjusting only for the district pairing (and not the village pairs) are also presented. Overall, practically-perfect balance in terms of exogenous variables such as household or individual characteristics is achieved, but there are a few differences in the AWC and AWW level characteristics and service delivery as reported by the AWWs. Almost all indicators related to home visits and growth monitoring were balanced as the magnitude of the group differences are of little practical significance, except for the growth monitoring related outcomes in Bihar. A few secondary outcomes were meaningfully different as well. However, such differences are expected when more than 100 covariates and outcome indicators are tested for balance. We also do not see a discernible pattern where control or intervention groups are consistently better or worse off than the other group. Considering the preponderance of highly similar distribution of variables, we infer that the matching resulted in exchangeable or balanced groups, and any residual confounding at the community level can be removed by controlling for the baseline outcomes.

DISCUSSION

The evaluation will provide evidence on whether and to what extent ICDS-CAS *mHealth* can improve health and nutrition service delivery beyond what is feasible with traditional non-technology-based approaches under the ISSNIP. Additionally, the analysis of a range of lower order outputs and outcomes can help us identify the pathways through which ICDS-CAS has worked, or the critical failure points.

For peer review only

The study faces a few limitations in identifying unbiased estimates of the program effects due to the nature of the intervention and constraints on the study design. First, confounding or selection bias cannot be theoretically ruled out in an observational study such as ours. While the matching procedure appears to be successful, it may not have removed all residual and unobserved confounding. Measurement biases including the Hawthorne effect[27] are possible because the outcomes are measured through interview recalls and observations. The external validity of the findings can be questioned because of the purposive sampling of states, pairing of districts, and the PSM-based sampling of villages does not result in a statistically representative sample of entire ICDS-CAS program area. Finally, as is the case with most large-scale programmes, ICDS-CAS implementation may be delayed and the planned follow-up period may not be adequate for the impacts to materialise.

While these limitations are common in observational studies, the study team has tried to minimize the risk to validity of the findings by reducing the observed pre-intervention imbalance using a large set of variables from Census 2011 for matching, controlling for *at least* the cluster level time-invariant confounders by using a repeated cross sectional design, measuring the primary outcomes at the beneficiary level who will be blinded to their intervention status in the study, measuring a large set of indicators as per the logic model to test whether ICDS-CAS is working through hypothesized pathways, and delaying the endline survey as much as possible before the intervention must be implemented in the control districts. The evaluation framework also includes other components which can assess the intervention using mixed methods approaches, and can help build confidence on the study findings.

Overall, this study will contribute to the evidence base on whether *mHealth* interventions can improve community health workers' efficiency and effectiveness. This is also a highly policy-relevant evaluation which can inform scale up of the intervention to potentially cover the entire country by 2020.

ETHICS AND DISSEMINATION

Study protocols have been reviewed and approved by institutional review boards at the University of California, Berkeley (Ref. No. 2016-08-9092), and the India-based Suraksha Independent Ethics Committee (Protocol No. 2016-08-9092). The trial is registered with The ISRCTN registry (https://doi.org/10.1186/ISRCTN83902145). The results will be published in peer reviewed journals and presented in conferences and dissemination meetings.

DATA SHARING STATEMENT

Deidentified data, questionnaires, and indicator construction sheet will be made publicly available after publication of the impact evaluation manuscript as per the funding and publication agreements with Bill and Melinda Gates foundation.

AUTHORS' CONTRIBUTIONS

DW and LF are the principal investigators on the grant from Bill and Melinda Gates Foundation. ND-S, LF, PM, HP, SPa, PSi, DW contributed to the study design and lead different sub-components of the study, DD commissioned the study and reviewed the study design, all authors were involved in protocol

For peer review only

development and finalising instruments. SN and SPa led the sampling. Data collection was mainly managed by SN, LG, RA, SM, AJ. SN, PN, ND-S and SPa conducted analyses and SN, LG, SPa developed the first draft of the manuscript. All authors reviewed, revised and approved the final manuscript.

FUNDING STATEMENT

This study is funded by Grant No. OPP1158231 from Bill and Melinda Gates Foundation to the University of California, San Francisco and University of California, Berkeley.

COMPETING INTERESTS STATEMENT

Diva Dhar is a Program Officer with the Measurement, Learning & Evaluation (MLE) team at the Bill & Melinda Gates Foundation (BMGF) India Country Office. BMGF has funded this study as well as the support to the scale-up of the ICDS-CAS programme. However, as part of the MLE team, Diva Dhar has had no role in the ICDS-CAS program design or implementation and was responsible for conceptualizing and commissioning the evaluation. She continuous to advise on study design, analysis and communication of findings to stakeholders. No competing interests declared by other authors.

For peer review only

1 International Institute for Population Sciences (IIPS) and ICF. National Family Health Survey (NFHS-4), 2015-16: India. Mumbai: : IIPS 2017. http://rchiips.org/NFHS/NFHS-4Reports/India.pdf (accessed 7 Mar 2018).

2 Rao N, Kaul V. India's integrated child development services scheme: challenges for scaling up. *Child Care Health Dev* 2018;**44**:31–40. doi:10.1111/cch.12531

3 Gragnolati M, Bredenkamp C, Gupta MD, *et al.* ICDS and Persistent Undernutrition: Strategies to Enhance the Impact. *Econ Polit Wkly* 2006;**41**:1193–201.

4 Kapil U, Pradhan R. Integrated Child Development Services scheme (ICDS) and its impact on nutritional status of children in India and recent initiatives. *Indian J Public Health* 1999;**43**:21.

5 Agarwal KN, Agarwal DK, Agarwal A, *et al.* Impact of the integrated child development services (ICDS) on maternal nutrition and birth weight in rural Varanasi. *Indian Pediatr* 2000;**37**:1321–7.

6 Tandon BN. Nutritional interventions through primary health care: impact of the ICDS projects in India. *Bull World Health Organ* 1989;**67**:77–80.

7 Kandpal E. Beyond Average Treatment Effects: Distribution of Child Nutrition Outcomes and Program Placement in India's ICDS. *World Dev* 2011;**39**:1410–21. doi:10.1016/j.worlddev.2010.12.013

8 India IF. Has the ICDS helped reduce stunting in India? Ideas India. http://www.ideasforindia.in/topics/human-development/has-the-icds-helped-reduce-stunting-in-india.html (accessed 6 Apr 2018).

9 Adhikari S, Bredenkamp C. Moving Towards An Outcomes-Oriented Approach to Nutrition Program Monitoring: The India ICDS Program. 2009.

10 Planning Commission Programme Evaluation Organisation. Evaluation Report on Integrated Child Development Services Vol.I. New Delhi: : Planning Commission, Government of India 2011. http://planningcommission.nic.in/reports/peoreport/peoevalu/peo_icds_v1.pdf (accessed 7 Mar 2018).

11 NITI Aayog Programme Evaluation Organisation. A Quick Evaluation Study of Anganwadis under ICDS. New Delhi: : NITI Aayog 2015. http://niti.gov.in/writereaddata/files/document_publication/report-awc.pdf (accessed 23 Apr 2018).

12 Evaluation of the Information and Communication Technology (ICT) Continuum of Care Services (CCS) Intervention in Bihar. Math. Policy Res. https://www.mathematica-mpr.com/our-publications-and-findings/publications/evaluation-of-the-information-and-communication-technology-ict-continuum-of-care-services-ccs (accessed 23 Mar 2018).

13 Lee SH, Nurmatov UB, Nwaru BI, *et al.* Effectiveness of mHealth interventions for maternal, newborn and child health in low– and middle–income countries: Systematic review and meta–analysis. *J Glob Health* 2016;**6**. doi:10.7189/jogh.06.010401

For peer review only

14 Barnett I, Gallegos JV. Using Mobile Phones for Nutrition Surveillance: A Review of Evidence. IDS 2013. https://opendocs.ids.ac.uk/opendocs/handle/123456789/2602 (accessed 11 Jun 2018).

15 Braun R, Catalani C, Wimbush J, *et al.* Community Health Workers and Mobile Technology: A Systematic Review of the Literature. *PLOS ONE* 2013;**8**:e65772. doi:10.1371/journal.pone.0065772

16 PM launches National Nutrition Mission, and pan India expansion of Beti Bachao Beti Padhao, at Jhunjhunu in Rajasthan. http://pib.nic.in/newsite/PrintRelease.aspx?relid=177166 (accessed 23 Mar 2018).

17 CAS Project Management Unit. Rollout of ICT-RTM Core Deck. 2018.

18 Rubin DB. Causal Inference Using Potential Outcomes. *J Am Stat Assoc* 2005;**100**:322–31. doi:10.1198/016214504000001880

19 Rubin DB. Estimating Causal Effects of Treatments in Randomized and Nonrandomized Studies. *J Educ Psychol* 1974.

20 Arnold BF, Khush RS, Ramaswamy P, *et al.* Causal inference methods to study nonrandomized, preexisting development interventions. *Proc Natl Acad Sci* 2010;**107**:22605–10. doi:10.1073/pnas.1008944107

21 Rubin D, R. Rosenbaum P. Constructing a Control Group Using Multivariate Matched Sampling Methods That Incorporate the Propensity Score. *Am Stat* 1985;**39**. doi:10.1080/00031305.1985.10479383

22 Stuart EA. Matching methods for causal inference: A review and a look forward. *Stat Sci Rev J Inst Math Stat* 2010;**25**:1–21. doi:10.1214/09-STS313

23 Caliendo Marco, Kopeinig Sabine. Some practical guidance for the implementation of propensity score matching. *J Econ Surv* 2008;**22**:31–72. doi:10.1111/j.1467-6419.2007.00527.x

24 Rosenbaum PR, Rubin DB. The central role of the propensity score in observational studies for causal effects. *Biometrika* 1983;**70**:41–55. doi:10.1093/biomet/70.1.41

25 Brady H, Collier D, S. Sekhon J. The Neyman-Rubin Model of Causal Inference and Estimation Via Matching Methods. *Oxf Handb Polit Methodol* Published Online First: 1 January 2008. doi:10.1093/oxfordhb/9780199286546.003.0011

26 Integrated Child Development Services: Home Visit Planner (Register 8). https://www.icds-wcd.nic.in/RevisedMIS/State%20Specific%20Formats%20in%20Local%20Languages/State%20Specific%20Revised%20MIS%20Formats/Goa/Reg-8-Home%20Visits%20Planner%20(English-Goa).pdf (accessed 8 Nov 2018).

27 Fernald DH, Coombs L, DeAlleaume L, *et al.* An Assessment of the Hawthorne Effect in Practice-based Research. *J Am Board Fam Med* 2012;**25**:83–6. doi:10.3122/jabfm.2012.01.110019

For peer review only

FIGURE LEGENDS

Figure 1. ICDS-CAS information flow from the Anganwadi Center to the Ministry of Women and Child Development. Solid lines correspond to interactions. Dotted lines correspond to data flow. ICDS-CAS, Integrated Child Development Services-Common Application Software; AWW, Anganwadi Worker; CDPO, Child Development Project Officer; MWCD, Ministry of Women and Child Development.

Figure 2. Logic Model of ICDS-CAS and Measurement of Outcomes. MWCD, Ministry of Women and Child Development; ICDS-CAS, Integrated Child Development Services-Common Application Software; AWW, Anganwadi Worker; CDPO, Child Development Project Officer; DPO, District Programme Officer; LS, Lady Supervisor.

Figure 3. Sampling of Study Participants from Madhya Pradesh and Bihar. ISSNIP, ICDS Systems Strengthening and Nutrition Improvement Programme; ICDS, Integrated Child Development Services; CAS, Common Application Software; AWC, Anganwadi Center; AWW, Anganwadi Worker.
- *Source*: Women and Child Development Department, Government of Madhya Pradesh (MIS) http://mpwcdmis.gov.in/(S(ee1m5bxnuzmixun00bsctvfj))/DataEntryAwc.aspx (Accessed 8 June 2018)
& - *Source*: Integrated Child Development Services, Government of Bihar http://www.icdsbih.gov.in/AnganwadiCenters.aspx?GL=16 (Accessed 8 June 2018)
***** - *Source*: Programme documentation from implementing agencies

Figure 4. Sampled Intervention and Comparison Districts. Green areas indicate intervention districts. Blue areas indicate comparison districts.

SUPPLEMENTARY FIGURE LEGEND

Supplementary Figure 1. Components of the ICDS-CAS Evaluation. ICDS-CAS, Integrated Child Development Services-Common Application Software; AWW, Anganwadi Worker; LS, Lady Supervisor; VHND, Village Health and Nutrition Day.

SUPPLEMENTARY TABLE LEGEND

Supplementary Table 1. Population, Health and Nutrition Related Indicators from National Family Health Survey (NFHS): India, Madhya Pradesh and Bihar, 2015-16.

Supplementary Table 2. Balance in Covariates at Anganwadi, Household and Individual Beneficiary levels.

Supplementary Table 3. Balance in Primary and Secondary Outcome Indicators.

For peer review only

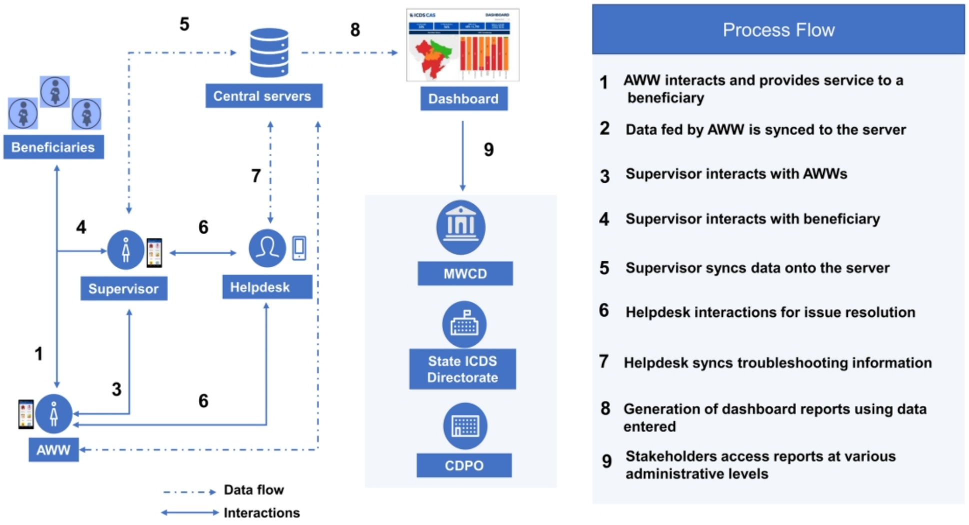

Figure 1. ICDS-CAS information flow from the Anganwadi Center to the Ministry of Women and Child Development.

190x107mm (300 x 300 DPI)

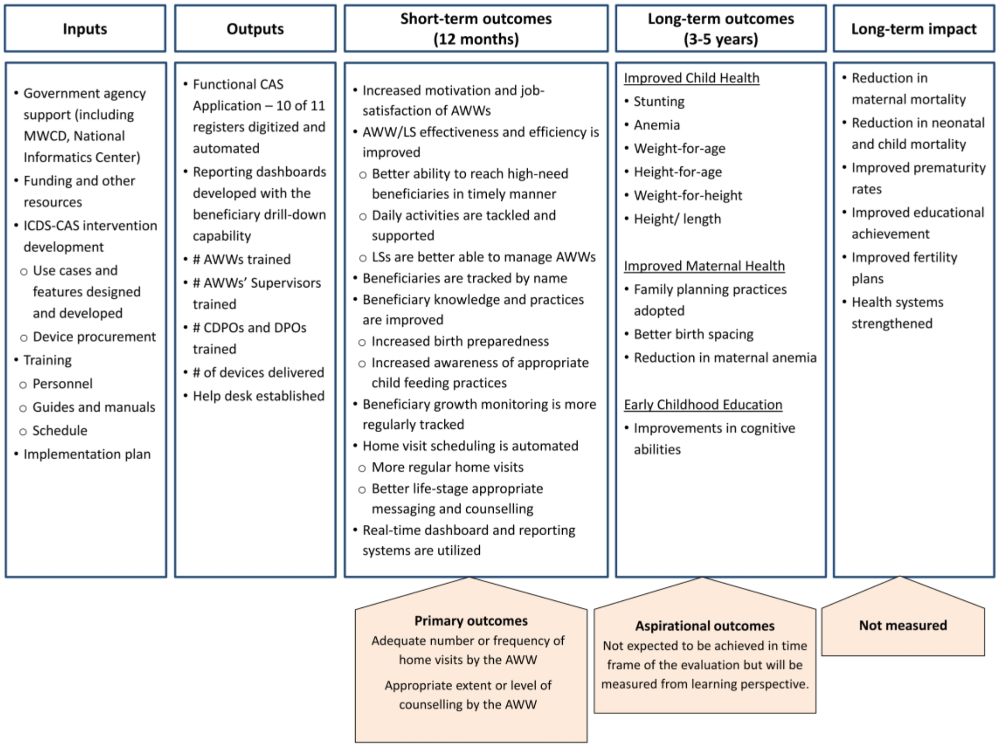

Logic Model of ICDS-CAS and Measurement of Outcomes.

1322x1017mm (96 x 96 DPI)

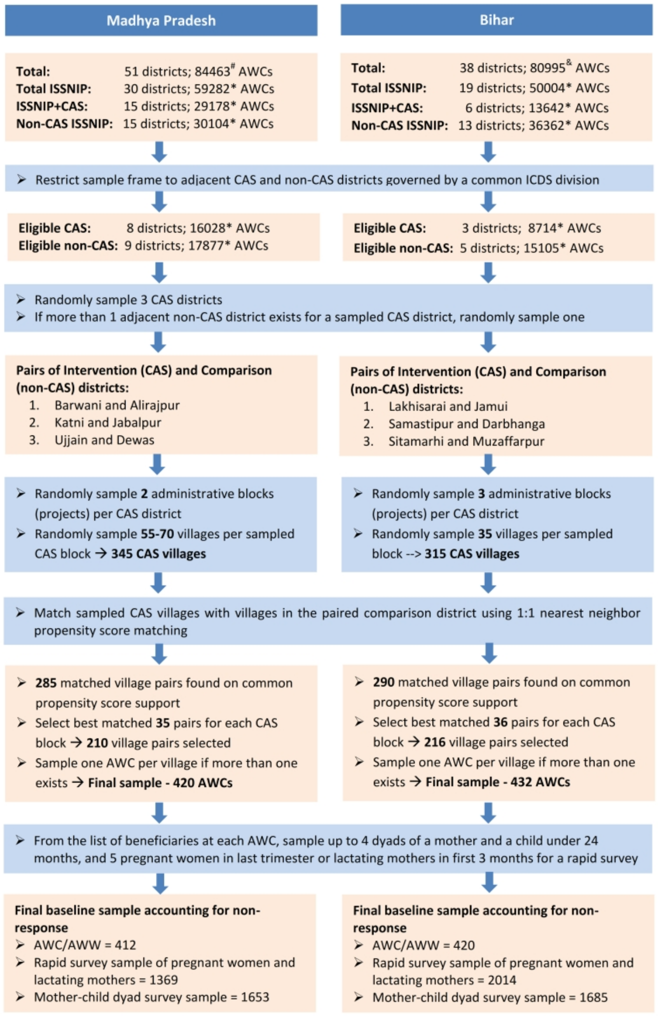

Figure 3. Sampling of Study Participants from Madhya Pradesh and Bihar.

297x420mm (300 x 300 DPI)

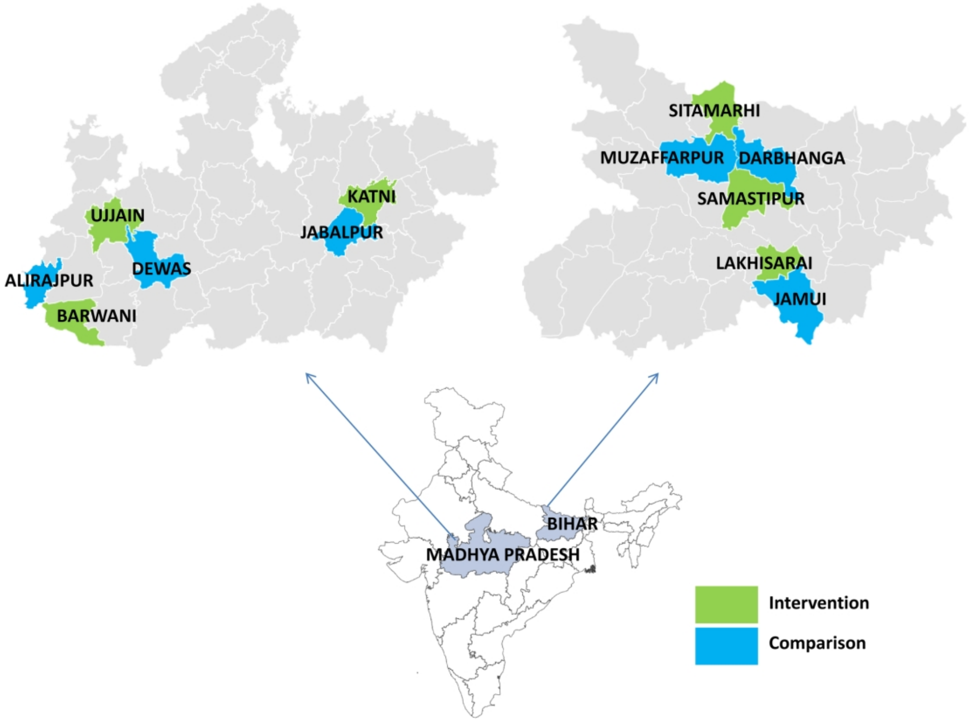

Figure 4. Sampled Intervention and Comparison Districts.

190x142mm (300 x 300 DPI)

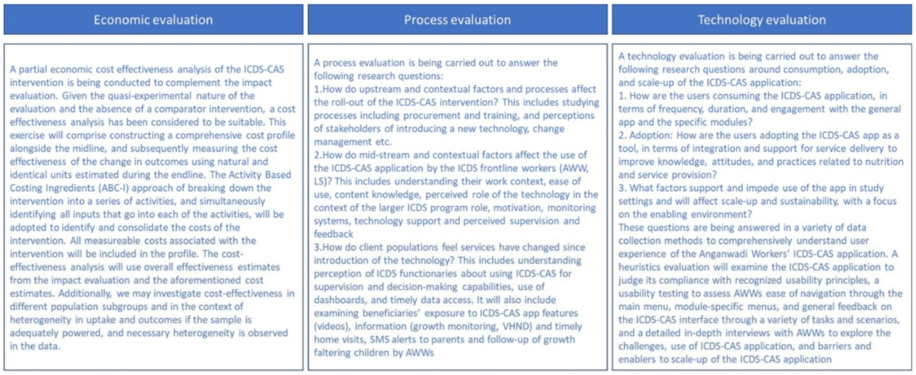

215x166mm (300 x 300 DPI)

Population, Health and Nutrition Related Indicators from National Family Health Survey (NFHS): India, Madhya Pradesh and Bihar, 2015-16

NFHS Indicators	India	Madhya Pradesh	Bihar
Sex ratio of the total population (females per 1,000 males)	1009	955	1075
Children under age 5 years whose birth was registered (%)	76.1	78.4	60.3
Households with electricity (%)	83.2	86.4	54.1
Households using clean fuel for cooking (%)	43.8	29.6	17.8
Households with an improved drinking-water source (%)	89.3	79.5	98.2
Households using improved sanitation facility (%)	36.7	19.4	20.7
Population (female) age 6 years and above who ever attended school (%)	68.8	64.0	56.9
Women with 10 or more years of schooling (%)	27.3	14.1	19.5
Women age 15-19 years who were ever pregnant (%)	9.2	8.6	12.8
Under-five mortality rate (U5MR)	56	69	60
Married women use of family planning methods (%)	51.7	51.3	22.6
Health worker ever talked to female non-users about family planning (%)	17.2	19.3	11.6
Mothers who consumed iron folic acid for 100+ days during pregnancy (%)	25.9	20.5	9.4
Mothers who had full antenatal care (%)	16.7	8.3	3.0
Institutional births (%)	75.1	76.4	62.7
Institutional births in public facility (%)	54.4	70.3	48.2
Children age 12-23 months fully immunized (%)	61.3	50.2	61.9
Children under age 6 months exclusively breastfed (%)	56.0	59.6	54.2
Total children age 6-23 months receiving an adequate diet (%)	8.8	6.0	7.4
Children under 5 years who are stunted (height-for-age) (%)	41.2	43.6	49.3
Children under 5 years who are wasted (weight-for-height) (%)	21.5	27.1	20.8
Children under 5 years who are underweight (weight-for-age) (%)	38.3	45.0	44.6
Women whose Body Mass Index (BMI) is below normal (<18.5 kg/m2) (%)	26.7	31.8	31.8
Children age 6-59 months who are anaemic (<11 g/dl) (%)	59.4	69.9	64.0
Pregnant women age 15-49 years who are anaemic (<11 g/dl) (%)	52.1	56.4	58.0

Source: NFHS-4 fact sheets for India, Madhya Pradesh and Bihar (http://rchiips.org/NFHS/factsheet_NFHS-4.shtml)

For peer review only - http://bmjopen.bmj.com/site/about/guidelines.xhtml

Balance in Covariates at Anganwadi, Household and Individual Beneficiary levels

Indicators	Bihar					Madhya Pradesh				
	N	Mean (Comp-arison)	Mean (Interv-ention)	Mean Diff. (Std. Err.)		N	Mean (Comp-arison)	Mean (Interv-ention)	Mean Diff. (Std. Err.)	
				District Pairs	Village Pairs				District Pairs	Village Pairs
**Respondent Characteristics**										
Average age in completed years	3143	24.31	24.90	0.587*** (0.18)	0.583*** (0.126)	2636	24.18	24.12	-0.065 (0.151)	-0.074 (0.118)
Average education level in years	3143	3.96	3.30	-0.663*** (0.231)	-0.695*** (0.17)	2636	4.55	4.92	0.353* (0.198)	0.35** (0.153)
% of respondents who work to earn income	3143	0.11	0.12	0.014 (0.015)	0.017 (0.011)	2636	0.45	0.44	-0.017 (0.02)	-0.018 (0.014)
% of respondents who have an Aadhaar card	3143	0.97	0.96	-0.015** (0.006)	-0.016*** (0.005)	2636	0.94	0.94	-0.002 (0.01)	-0.004 (0.008)
% of respondents who own a bank account	3143	0.77	0.80	0.029* (0.017)	0.026** (0.013)	2636	0.78	0.81	0.026 (0.019)	0.024* (0.014)
% of respondents who are currently married	3143	1.00	1.00	-0.001 (0.002)	-0.001 (0.002)	2636	0.99	0.99	0 (0.003)	0.001 (0.002)
% of respondents who were residing in their permanent marital home at the time of survey	3143	0.88	0.89	0.012 (0.013)	0.012 (0.009)	2636	0.92	0.9	-0.019 (0.012)	-0.017* (0.009)
% of respondents who were residing in their permanent maternal home at the time of survey	3143	0.02	0.01	-0.011** (0.005)	-0.011*** (0.004)	2636	0.01	0.01	0.002 (0.004)	0.002 (0.003)
**Household Socio-economics**										
% of households which belong to Scheduled Castes or Scheduled Tribe categories	3143	0.35	0.38	0.03 (0.03)	0.031 (0.023)	2636	0.56	0.5	-0.061** (0.028)	-0.06*** (0.021)

For peer review only

Indicators	Bihar					Madhya Pradesh				
	N	Mean (Comp-arison)	Mean (Interv-ention)	Mean Diff. (Std. Err.)		N	Mean (Comp-arison)	Mean (Interv-ention)	Mean Diff. (Std. Err.)	
				District Pairs	Village Pairs				District Pairs	Village Pairs
% of households which belong to Below-Poverty-Line category	3143	**0.58**	**0.62**	0.039* (0.022)	0.043** (0.017)	2636	0.63	0.66	0.024 (0.022)	0.023 (0.017)
% of households with pucca construction	3143	0.38	0.37	-0.007 (0.024)	-0.009 (0.017)	2636	0.26	0.3	0.036 (0.024)	0.033* (0.018)
% of households with fewer than two rooms for sleeping	3143	**0.34**	**0.41**	0.068*** (0.022)	0.067*** (0.016)	2636	0.39	0.41	0.017 (0.023)	0.022 (0.017)
% of households that own agricultural land	3143	0.47	0.42	-0.051** (0.026)	-0.055*** (0.019)	2636	0.76	0.74	-0.021 (0.022)	-0.019 (0.017)
% of households that mainly use improved fuels for cooking	3143	0.17	0.15	-0.024 (0.017)	-0.024* (0.013)	2636	0.1	0.14	0.033** (0.016)	0.032*** (0.011)
% of households with improved drinking water source on premises	3143	**0.61**	**0.55**	-0.053** (0.025)	-0.055*** (0.02)	2636	0.14	0.14	0.001 (0.018)	0.004 (0.013)
% of households with a functional toilet	3143	**0.24**	**0.39**	0.156*** (0.025)	0.152*** (0.019)	2636	**0.52**	**0.46**	-0.061** (0.029)	-0.063*** (0.022)
% of households with any member practicing open defecation	3143	**0.81**	**0.70**	-0.107*** (0.023)	-0.105*** (0.018)	2636	**0.62**	**0.71**	0.086*** (0.027)	0.086*** (0.02)
% of households with piped water	3143	0.01	0.01	0.004 (0.005)	0.004 (0.004)	2636	0.08	0.07	-0.006 (0.017)	-0.006 (0.013)
% of households with a handwashing facility	3143	0.69	0.66	-0.027 (0.029)	-0.032 (0.021)	2636	0.62	0.65	0.025 (0.03)	0.025 (0.022)
**Anganwadi Center (AWC) Coverage**										
Average population in catchment area	419	1111.70	1116.33	4.633 (28.769)	-2.274 (28.894)	406	730.32	857.24	124.258*** (29.916)	123.823*** (30.838)
Average number of families in catchment area	415	172.45	185.66	13.555** (5.458)	12.706** (5.66)	406	126	153.69	26.827*** (5.223)	26.54*** (5.39)

Indicators	Bihar					Madhya Pradesh				
	N	Mean (Comp-arison)	Mean (Interv-ention)	Mean Diff. (Std. Err.)		N	Mean (Comp-arison)	Mean (Interv-ention)	Mean Diff. (Std. Err.)	
				District Pairs	Village Pairs				District Pairs	Village Pairs
Average number of children aged 0-5 months in catchment area	403	10.56	11.10	0.551 (1.12)	0.606 (1.194)	404	5.64	9.32	3.697*** (1.232)	3.655*** (1.269)
Average number of children aged 6-12 months in catchment area	393	17.64	17.26	-0.307 (1.559)	-0.283 (1.757)	400	7.62	9.58	1.973*** (0.698)	1.984*** (0.75)
Average number of children aged 12-24 months in catchment area	387	27.78	27.80	0.208 (2.256)	0.017 (2.446)	398	15	19.98	5.011*** (1.085)	5.34*** (1.164)
Average number of pregnant women in catchment area	417	12.30	12.36	0.053 (0.458)	0.068 (0.464)	406	7.39	7.71	0.306 (0.576)	0.237 (0.604)
Average number of lactating women in catchment area	417	12.05	11.10	-0.948* (0.513)	-0.976* (0.508)	406	5.86	7.36	1.503*** (0.405)	1.444*** (0.43)
**Anganwadi Center (AWC) Characteristics**										
% AWCs with pucca construction	420	0.55	0.52	-0.033 (0.047)	-0.033 (0.047)	412	0.8	0.85	0.04 (0.037)	0.039 (0.039)
% AWCs with an Anganwadi Helper	420	0.91	0.91	-0.004 (0.028)	-0.005 (0.028)	412	0.96	0.97	0.005 (0.019)	0.005 (0.019)
% AWCs with drinking water on the premises	420	0.43	0.46	0.029 (0.049)	0.029 (0.049)	412	0.68	0.58	-0.098** (0.046)	-0.108** (0.048)
% AWCs with a functional toilet on the premises	420	0.11	0.18	0.07** (0.035)	0.062* (0.034)	412	0.39	0.29	-0.1** (0.045)	-0.103** (0.045)
% AWCs with electricity	420	0.16	0.17	0.003 (0.036)	0.005 (0.037)	412	0.21	0.15	-0.055 (0.038)	-0.054 (0.038)
% AWCs with Salter scales to weigh children	420	0.59	0.74	0.151*** (0.044)	0.148*** (0.044)	412	0.96	0.91	-0.048** (0.024)	-0.049* (0.025)
% AWCs with the new WHO Growth Monitoring charts	420	0.28	0.30	0.026 (0.044)	0.024 (0.048)	412	0.69	0.58	-0.108** (0.046)	-0.113** (0.048)
**Anganwadi Worker (AWW) Characteristics**										

For peer review only

Indicators	Bihar					Madhya Pradesh				
	N	Mean (Comp-arison)	Mean (Interv-ention)	Mean Diff. (Std. Err.)		N	Mean (Comp-arison)	Mean (Interv-ention)	Mean Diff. (Std. Err.)	
				District Pairs	Village Pairs				District Pairs	Village Pairs
Average age in completed years	420	39.11	37.54	-1.565** (0.744)	-1.555** (0.756)	412	39.45	40.57	1.124 (0.868)	1.069 (0.888)
Average education level in years	420	11.53	11.81	0.283 (0.201)	0.278 (0.203)	412	10.12	10.86	0.739** (0.334)	0.75** (0.336)
% AWWs who belong to Scheduled Castes or Scheduled Tribe categories	420	0.17	0.21	0.041 (0.038)	0.043 (0.039)	412	0.53	0.43	-0.102*** (0.039)	-0.108** (0.042)
% AWWs who own a bank account	420	0.99	1.00	0.01 (0.009)	0.01 (0.01)	412	1	1	0.005 (0.005)	0.005 (0.005)
% AWWs who are currently married	420	0.93	0.96	0.024 (0.022)	0.024 (0.023)	412	0.82	0.87	0.05 (0.036)	0.049 (0.038)
% AWWs who are presently engaged in any other work to earn additional income	420	0.01	0.06	0.042** (0.018)	0.043** (0.017)	412	0.03	0.12	0.087*** (0.026)	0.088*** (0.026)
% AWWs who received job training on joining	420	0.99	0.97	-0.014 (0.014)	-0.014 (0.014)	382	0.95	0.93	-0.015 (0.025)	-0.017 (0.028)
% AWWs who report ownership of a smart phone	420	0.03	0.93	0.895*** (0.021)	0.895*** (0.021)	412	0.23	0.94	0.713*** (0.034)	0.711*** (0.036)
**% AWWs who report providing the following services in the last calendar month**										
Take Home Ration to pregnant women, lactating women, and children < 3 years	420	0.70	0.19	-0.509*** (0.041)	-0.507*** (0.041)	412	0.98	0.96	-0.014 (0.017)	-0.015 (0.018)
Home Visits	420	0.86	0.89	0.034 (0.031)	0.033 (0.031)	412	0.98	0.98	0 (0.014)	0 (0.014)
Iron supplements to pregnant women	420	0.13	0.30	0.174*** (0.039)	0.172*** (0.04)	412	0.87	0.87	0.006 (0.033)	0 (0.033)
Iron supplements to children aged 6 months-2 years	420	0.91	0.96	0.048** (0.024)	0.048** (0.023)	412	0.98	0.93	-0.048** (0.02)	-0.049** (0.019)

For peer review only

Indicators	Bihar					Madhya Pradesh				
	N	Mean (Comp-arison)	Mean (Interv-ention)	Mean Diff. (Std. Err.)		N	Mean (Comp-arison)	Mean (Interv-ention)	Mean Diff. (Std. Err.)	
				District Pairs	Village Pairs				District Pairs	Village Pairs
Vaccination	420	0.16	0.19	0.031 (0.036)	0.029 (0.037)	412	0.6	0.7	0.096** (0.047)	0.098** (0.047)
Vitamin A supplements	420	0.06	0.06	-0.005 (0.023)	-0.005 (0.024)	412	0.71	0.69	-0.021 (0.044)	-0.02 (0.047)
Growth monitoring	420	0.44	0.52	0.076 (0.047)	0.072 (0.045)	412	0.96	0.95	-0.009 (0.021)	-0.01 (0.022)
Pre-school education to children aged 3 years and above	420	0.99	1.00	0.014* (0.008)	0.014* (0.008)	412	0.99	0.98	-0.01 (0.012)	-0.01 (0.012)
**AWW Knowledge**										
% AWWs who knew at least half the correct messages and checks to be given to women in last trimester of pregnancy	420	0.26	0.26	-0.008 (0.041)	-0.005 (0.041)	412	0.29	0.25	-0.041 (0.043)	-0.044 (0.043)
% AWWs who knew at least half the correct messages and checks to be given to women who have recently delivered a child	420	0.09	0.09	0.004 (0.026)	0.005 (0.028)	412	0.12	0.06	-0.059** (0.026)	-0.059** (0.026)
% AWWs who knew at least half the correct messages and checks for women with a child aged 6 months	420	0.13	0.16	0.031 (0.032)	0.033 (0.034)	412	0.23	0.16	-0.069* (0.037)	-0.069* (0.039)
% AWWs with knowledge* on Pregnancy care and Birth Preparedness, as per the technical quiz	420	0.77	0.66	-0.107** (0.042)	-0.11*** (0.04)	412	0.64	0.65	0.004 (0.046)	0.01 (0.045)
% AWWs with knowledge on New Born Care in the technical quiz	420	0.33	0.26	-0.065 (0.043)	-0.067 (0.043)	412	0.34	0.38	0.045 (0.047)	0.049 (0.046)

For peer review only

Indicators	Bihar					Madhya Pradesh				
	N	Mean (Comp-arison)	Mean (Interv-ention)	Mean Diff. (Std. Err.)		N	Mean (Comp-arison)	Mean (Interv-ention)	Mean Diff. (Std. Err.)	
				District Pairs	Village Pairs				District Pairs	Village Pairs
% AWWs with knowledge on Family Planning in the technical quiz	420	0.84	0.88	0.039 (0.032)	0.038 (0.034)	412	0.86	0.9	0.04 (0.031)	0.039 (0.033)
% AWWs with knowledge on Breastfeeding in the technical quiz	420	0.85	0.85	0.001 (0.034)	0 (0.033)	412	0.84	0.87	0.031 (0.034)	0.029 (0.035)
% AWWs with knowledge on Complementary feeding in the technical quiz	420	0.99	0.99	0 (0.01)	0 (0.01)	412	0.99	0.97	-0.019 (0.015)	-0.02 (0.016)
% AWWs who correctly answered at least half the questions in the Growth Monitoring quiz	420	0.45	0.45	-0.001 (0.042)	-0.005 (0.042)	412	0.68	0.72	0.042 (0.044)	0.044 (0.044)

For peer review only

Balance in Primary and Secondary Outcome Indicators

Indicators	Bihar					Madhya Pradesh				
	N	Mean (Comp-arison)	Mean (Interv-ention)	Mean Diff. (Std. Err.)		N	Mean (Comp-arison)	Mean (Interv-ention)	Mean Diff. (Std. Err.)	
				District Pairs	Village pairs				District Pairs	Village pairs
**Primary Outcomes**										
% of beneficiaries with 0-6m child who report at least two home visits by Anganwadi Workers (AWW) during their last trimester of pregnancy	810	0.05	0.08	0.026 (0.018)	0.031** (0.015)	757	0.2	0.23	0.036 (0.032)	0.035 (0.028)
% of beneficiaries with 0-6m child who received at least half the messages as per Integrated Child Development Scheme (ICDS) guidelines during their last trimester of pregnancy	810	0.08	0.12	0.038* (0.022)	0.039** (0.019)	757	0.2	0.23	0.037 (0.031)	0.027 (0.027)
% beneficiaries with 0-6m child who received any visit from AWW within 24 hours after birth	810	0.06	0.05	-0.016 (0.017)	-0.018 (0.015)	757	0.07	0.1	0.032 (0.021)	0.04** (0.018)
% beneficiaries with 0-6m child who received at least 2 visits within the first week of birth	810	0.03	0.03	0.002 (0.012)	0.001 (0.01)	757	0.04	0.05	0.003 (0.016)	0.004 (0.014)
% beneficiaries with 0-6m child who received appropriate number of visits as per ICDS guidelines within first six months of birth	810	0.02	0.03	0.014 (0.012)	0.013 (0.011)	757	0.04	0.04	0 (0.016)	0.002 (0.013)
% beneficiaries with 6-12m child who received appropriate number of visits as per ICDS guidelines since their child turned 6 months	444	0.03	0.05	0.023 (0.018)	0.025 (0.018)	465	0.14	0.12	-0.027 (0.031)	-0.03 (0.033)

For peer review only

Indicators	Bihar					Madhya Pradesh				
	N	Mean (Comp-arison)	Mean (Interv-ention)	Mean Diff. (Std. Err.)		N	Mean (Comp-arison)	Mean (Interv-ention)	Mean Diff. (Std. Err.)	
				District Pairs	Village pairs				District Pairs	Village pairs
% of beneficiaries with 6-12m child who received at least half the messages as per ICDS guidelines since their child turned 6 months	444	0	0.01	0.005 (0.008)	0.005 (0.008)	465	0.07	0.05	-0.019 (0.022)	-0.019 (0.022)
% beneficiaries with 12-24m child who received at least 2 visits since their child turned 12 months	431	0.97	0.96	-0.009 (0.018)	-0.011 (0.017)	431	0.94	0.91	-0.035 (0.025)	-0.03 (0.028)
% beneficiaries with child 0-24m who reported that their child was weighed by AWW in the last 3 months	1685	0.16	0.24	0.085*** (0.024)	0.085*** (0.021)	1653	0.54	0.6	0.057** (0.028)	0.057*** (0.022)
% beneficiaries with child 0-24m who reported that AWW discussed their child's weight and nutritional status in the last 3 months	1685	0.11	0.17	0.063*** (0.02)	0.063*** (0.017)	1653	0.37	0.39	0.021 (0.029)	0.021 (0.021)
**Secondary Outcomes**										
**Knowledge about Pregnancy care, Birth preparedness, Health and Nutrition**										
% beneficiaries with 0-6m child who are aware of at least half of the things a pregnant woman should do to take care of herself and baby during pregnancy	810	0.57	0.52	-0.052 (0.04)	-0.047 (0.034)	757	0.59	0.53	-0.06 (0.037)	-0.06** (0.03)
% beneficiaries with 0-6m child who are aware of at least half of the infant danger signs	810	0.04	0.03	-0.016 (0.014)	-0.013 (0.012)	757	0.02	0.06	0.04*** (0.015)	0.041*** (0.013)

For peer review only

Indicators	Bihar					Madhya Pradesh				
	N	Mean (Comp-arison)	Mean (Interv-ention)	Mean Diff. (Std. Err.)		N	Mean (Comp-arison)	Mean (Interv-ention)	Mean Diff. (Std. Err.)	
				District Pairs	Village pairs				District Pairs	Village pairs
% of beneficiaries with 0-6m child who know how soon an infant should be breastfed after birth of the child	810	0.7	0.72	0.022 (0.033)	0.025 (0.027)	757	0.67	0.72	0.055 (0.034)	0.055* (0.029)
% beneficiaries with 0-6m child who know that infants should not be given water when the infant is less than 6m old	810	0.43	0.35	-0.086** (0.036)	-0.091*** (0.03)	757	0.35	0.37	0.023 (0.036)	0.032 (0.031)
**Services received during pregnancy**										
% currently pregnant women who registered their pregnancy with either Accredited Social Health Activist (ASHA), Auxiliary Nurse Midwife (ANM), AWW, or Govt. Center	1458	0.95	0.97	0.017 (0.011)	0.021** (0.009)	983	0.97	0.99	0.01 (0.009)	0.008 (0.007)
% currently pregnant women who received ANY Antenatal Care (ANC) check-up during their pregnancy	1458	0.83	0.85	0.024 (0.023)	0.029 (0.02)	983	0.93	0.98	0.051*** (0.015)	0.038*** (0.011)
% currently pregnant women who report attending at least one Villahe Health and Nutrition Day (VHND) during their pregnancy	1458	0.47	0.5	0.029 (0.035)	0.022 (0.028)	983	0.65	0.57	-0.089** (0.036)	-0.086*** (0.033)
**Ante-natal Care (ANC)**										
% beneficiaries with 0-24m child who received at least 4 ANC check-ups during pregnancy	1685	0.25	0.2	-0.05** (0.023)	-0.05*** (0.017)	1653	0.52	0.51	-0.003 (0.028)	-0.005 (0.022)
% beneficiaries with 0-24m child who consumed Iron Folic Acid for more than 90 days during pregnancy	1685	0.12	0.12	0.001 (0.017)	0.001 (0.013)	1653	0.54	0.49	-0.054** (0.027)	-0.055** (0.022)

For peer review only

Indicators	Bihar					Madhya Pradesh				
	N	Mean (Comparison)	Mean (Intervention)	Mean Diff. (Std. Err.)		N	Mean (Comparison)	Mean (Intervention)	Mean Diff. (Std. Err.)	
				District Pairs	Village pairs				District Pairs	Village pairs
% beneficiaries with 0-24m child who received 2 Tetanus Toxoid (TT) injections during pregnancy	1685	0.84	0.81	-0.033 (0.02)	-0.032** (0.016)	1653	0.81	0.87	0.061*** (0.02)	0.061*** (0.016)
% beneficiaries with 0-24m child who delivered at a health facility	1685	0.73	0.65	-0.079*** (0.028)	-0.079*** (0.021)	1653	0.81	0.81	0.008 (0.022)	0.007 (0.016)
**Beneficiary practices post-delivery**										
% beneficiaries with 0-24m child who breastfed their child within an hour of birth	1685	0.76	0.72	-0.04 (0.025)	-0.04** (0.018)	1653	0.72	0.75	0.024 (0.026)	0.022 (0.019)
% beneficiaries with 0-24m child who had applied nothing to the cord after birth	1685	0.57	0.59	0.025 (0.028)	0.026 (0.021)	1653	0.6	0.6	0.006 (0.027)	0.007 (0.021)
**AWW Home Visits**										
% currently pregnant women who report ANY home visits by AWW during pregnancy	1458	0.23	0.3	0.065** (0.028)	0.057** (0.023)	983	0.39	0.42	0.025 (0.039)	0.012 (0.034)
% currently pregnant women who report that the AWW discussed or checked their Iron Folic Acid intake during a home visit	1458	0.1	0.19	0.091*** (0.024)	0.085*** (0.019)	983	0.36	0.39	0.034 (0.038)	0.024 (0.033)
% currently pregnant women who report that the AWW asked about ANC check-ups, immunization, blood tests, etc. during a home visit	1458	0.13	0.19	0.061** (0.026)	0.047** (0.021)	983	0.31	0.37	0.059* (0.035)	0.053* (0.031)

For peer review only

Indicators	Bihar					Madhya Pradesh				
	N	Mean (Comp-arison)	Mean (Interv-ention)	Mean Diff. (Std. Err.)		N	Mean (Comp-arison)	Mean (Interv-ention)	Mean Diff. (Std. Err.)	
				District Pairs	Village pairs				District Pairs	Village pairs
% beneficiaries with 0-6m child who report that AWW discussed child's birth weight and health status during home visit	810	0.04	0.06	0.021 (0.016)	0.021 (0.014)	757	0.12	0.13	0.013 (0.025)	0.012 (0.021)
% beneficiaries with 6-24m child who reported AWWs discussing child's weight and adequacy of growth during home visit	875	0.03	0.07	0.042*** (0.016)	0.042*** (0.014)	896	0.21	0.14	-0.064** (0.028)	-0.063*** (0.023)
**Family Planning**										
% beneficiaries with 0-24m child who received advice on family planning needs, methods, resources	1685	0.02	0.04	0.023*** (0.008)	0.023*** (0.007)	1653	0.08	0.09	0.004 (0.015)	0.004 (0.011)
% beneficiaries with 0-24m child who is currently using family planning	1685	0.3	0.27	-0.023 (0.025)	-0.023 (0.019)	1653	0.37	0.34	-0.023 (0.025)	-0.023 (0.019)
**Take Home Ration (THR)**										
% currently pregnant beneficiaries who received THR from the AWC during pregnancy	1458	0.53	0.4	-0.131*** (0.034)	-0.127*** (0.027)	983	0.9	0.88	-0.026 (0.022)	-0.023 (0.019)
% beneficiaries with 6-24m child who received THR in the first 6 months after birth of child	875	0.71	0.58	-0.129*** (0.035)	-0.123*** (0.03)	896	0.91	0.92	0.007 (0.019)	0.008 (0.015)
% beneficiaries with 6-24m child who received THR for child > 6m	875	0.5	0.43	-0.068* (0.036)	-0.06** (0.03)	896	0.75	0.77	0.026 (0.031)	0.029 (0.025)
% beneficiaries with 0-24m child who consumed entire THR themselves during pregnancy	1685	0.23	0.19	-0.038 (0.023)	-0.038** (0.018)	1653	0.33	0.39	0.063** (0.026)	0.062*** (0.02)
**Infant and Young Child Feeding (IYCF) - Knowledge and Practices**										

For peer review only

Indicators	Bihar					Madhya Pradesh				
	N	Mean (Comp-arison)	Mean (Interv-ention)	Mean Diff. (Std. Err.)		N	Mean (Comp-arison)	Mean (Interv-ention)	Mean Diff. (Std. Err.)	
				District Pairs	Village pairs				District Pairs	Village pairs
% beneficiaries with 6-12m child who know the right time to start complementary feeding	444	0.95	0.93	-0.021 (0.023)	-0.02 (0.022)	465	0.88	0.9	0.015 (0.029)	0.01 (0.027)
% infants 0-5m of age who received only breastmilk during the previous day	810	0.6	0.51	-0.087*** (0.033)	-0.088*** (0.028)	757	0.46	0.5	0.032 (0.034)	0.038 (0.03)
% infants 6-8m of age who received solid, semi-solid or soft foods during the previous day	266	0.53	0.55	0.016 (0.06)	0.034 (0.088)	264	0.56	0.62	0.051 (0.06)	0.137 (0.094)
% children 6-24m of age who received foods from >= 4 food groups during the previous day	875	0.12	0.15	0.038* (0.023)	0.04** (0.019)	896	0.17	0.22	0.052* (0.027)	0.054** (0.022)
**Growth Monitoring**										
% beneficiaries with 0-6m child who were aware that growth monitoring for children was one of the services at AWCs	810	0.1	0.14	0.039 (0.026)	0.037* (0.021)	757	0.3	0.21	-0.093*** (0.033)	-0.099*** (0.027)
% beneficiaries with 0-24m child who reported that child birth weight as available	1685	0.67	0.58	-0.08*** (0.028)	-0.081*** (0.021)	1653	0.79	0.81	0.02 (0.021)	0.02 (0.015)
% beneficiaries with 0-24m child whose child's birth weight was verified from Mother-Child Protection (MCP)/Health card	1685	0.16	0.15	-0.011 (0.019)	-0.011 (0.014)	1653	0.24	0.29	0.045* (0.024)	0.043** (0.019)
**Diet and Food security**										

For peer review only

Indicators	Bihar					Madhya Pradesh				
	N	Mean (Comp-arison)	Mean (Interv-ention)	Mean Diff. (Std. Err.)		N	Mean (Comp-arison)	Mean (Interv-ention)	Mean Diff. (Std. Err.)	
				District Pairs	Village pairs				District Pairs	Village pairs
% mothers of 0-24m children who have consumed at least five out of ten defined food groups the previous day or night	1685	0.16	0.18	0.015 (0.021)	0.015 (0.016)	1653	0.14	0.16	0.015 (0.021)	0.016 (0.016)
% households which are food secure[1]	1685	0.79	0.7	-0.086*** (0.023)	-0.086*** (0.018)	1653	0.76	0.75	-0.01 (0.023)	-0.011 (0.018)
**Immunization**										
% beneficiaries whose child aged 0-24m ever received any immunizations	1685	0.97	0.97	0 (0.009)	0 (0.007)	1653	0.97	0.98	0.01 (0.008)	0.009 (0.006)
% beneficiaries with 0-24m child who are aware that AWW records details of immunization	1685	0.26	0.3	0.039 (0.025)	0.039** (0.018)	1653	0.32	0.31	-0.016 (0.027)	-0.016 (0.021)
% beneficiaries with 0-24m child who reported that they had MCP cards	1685	0.86	0.86	-0.005 (0.018)	-0.005 (0.014)	1653	0.74	0.91	0.174*** (0.022)	0.175*** (0.017)
% beneficiaries with 0-24m child whose MCP cards were available at home or at Anganwadi Center, and were at least partially filled	1685	0.63	0.65	0.021 (0.025)	0.021 (0.02)	1653	0.54	0.69	0.15*** (0.027)	0.149*** (0.021)
% beneficiaries whose child aged 12-24m is fully immunized	431	0.31	0.35	0.041 (0.045)	0.043 (0.044)	431	0.31	0.4	0.096** (0.044)	0.097** (0.046)
% beneficiaries whose child aged 12-24m is fully immunized (among the subset of beneficiaries with available MCP cards)	293	0.43	0.52	0.083 (0.059)	0.059 (0.082)	268	0.53	0.57	0.069 (0.062)	0.132 (0.096)

[1] Food and Agricultural Organization (FAO) "Food Insecurity Experience Scale Survey Module (FIES-SM) – Household Referenced" (http://www.fao.org/3/a-bl404e.pdf)

For peer review only
